# Artificial targeting of autophagy components to mitochondria reveals both conventional and unconventional mitophagy pathways

**DOI:** 10.1080/15548627.2024.2395149

**Published:** 2024-08-23

**Authors:** Katharina C. Lorentzen, Alan R. Prescott, Ian G. Ganley

**Affiliations:** a MRC Protein Phosphorylation and Ubiquitylation Unit, University of Dundee, Dundee, UK; b Dundee Imaging Facility, School of Life Sciences, University of Dundee, Dundee, UK

**Keywords:** Atg8, ATG16L1, nanobody, targeted organelle degradation, ULK1

## Abstract

Macroautophagy/autophagy enables lysosomal degradation of a diverse array of intracellular material. This process is essential for normal cellular function and its dysregulation is implicated in many diseases. Given this, there is much interest in understanding autophagic mechanisms of action in order to determine how it can be best targeted therapeutically. In mitophagy, the selective degradation of mitochondria via autophagy, mitochondria first need to be primed with signals that allow the recruitment of the core autophagy machinery to drive the local formation of an autophagosome around the target mitochondrion. To determine how the recruitment of different core autophagy components can drive mitophagy, we took advantage of the *mito*-QC mitophagy assay (an outer mitochondrial membrane-localized tandem mCherry-GFP tag). By tagging autophagy proteins with an anti-mCherry (or anti-GFP) nanobody, we could recruit them to mitochondria and simultaneously monitor levels of mitophagy. We found that targeting ULK1, ATG16L1 and the different Atg8-family proteins was sufficient to induce mitophagy. Mitochondrial recruitment of ULK1 and the Atg8-family proteins induced a conventional mitophagy pathway, requiring RB1CC1/FIP200, PIK3C3/VPS34 activity and ATG5. Surprisingly, the mitophagy pathway upon recruitment of ATG16L1 proceeded independently of ATG5, although it still required RB1CC1 and PIK3C3/VPS34 activity. In this latter pathway, mitochondria were alternatively delivered to lysosomes via uptake into early endosomes.

**Abbreviation:** aGFP: anti-GFP nanobody; amCh: anti-mCherry nanobody; ATG: autophagy related; ATG16L1: autophagy related 16 like 1; AUTAC/AUTOTAC: autophagy-targeting chimera; BafA1: bafilomycin A_1_; CALCOCO2/NDP52: calcium binding and coiled-coil domain 2; CCCP: carbonyl cyanide m-chlorophenylhydrazone; COX4/COX IV: cytochrome c oxidase subunit 4; DFP: deferiprone; DMSO: dimethyl sulfoxide; GABARAP: GABA type A receptor-associated protein; GABARAPL1: GABA type A receptor associated protein like 1; HSPD1/HSP60: heat shock protein family D (Hsp60) member 1; HRP: horseradish peroxidase; HTRA2/OMI: HtrA serine peptidase 2; IB: immunoblotting; IF: immunofluorescence; KO: knockout; LAMP1: lysosomal associated membrane protein 1; LIR: LC3-interacting region; MAP1LC3/LC3: microtubule associated protein 1 light chain 3; MEF: mouse embryonic fibroblast; NBR1: NBR1 autophagy cargo receptor; OMM: outer mitochondrial membrane; OPA1: OPA1 mitochondrial dynamin like GTPase; OPTN: optineurin; (D)PBS: (Dulbecco’s) phosphate-buffered saline; PD: Parkinson disease; PFA: paraformaldehyde; POI: protein of interest; PtdIns3K: class III phosphatidylinositol 3-kinase; PtdIns3P: phosphatidylinositol-3-phosphate; RAB: RAB, member RAS oncogene family; RB1CC1/FIP200: RB1 inducible coiled-coil 1; SQSTM1: sequestosome 1; TAX1BP1: Tax1 binding protein 1; ULK: unc-51 like autophagy activating kinase 1; VPS: vacuolar protein sorting; WIPI: WD repeat domain, phosphoinositide interacting.

## Introduction

Targeted protein degradation using Proteolysis Targeting Chimera/PROTAC technology is emerging as a new strategy to study biological functions as well as a promising new therapeutic approach to target previously undruggable proteins in diseases. Here, small molecules recruit E3 ubiquitin ligases to target proteins to mediate their proteasomal turnover [1]. Likewise, evidence suggests that small molecules could lead to the recruitment of autophagy components to a target, resulting in its lysosomal degradation via the autophagic pathway [2–8]. Autophagic-lysosomal degradation has the advantage over proteasomal turnover in that also non-protein targets can be degraded, including whole organelles.

Autophagy is a catabolic process in eukaryotic cells that functions to deliver intracellular material, including organelles, into lysosomes for degradation. In canonical macroautophagy, a phagophore membrane emerges that expands around cargo to form a double-membraned autophagosome that then fuses with late endosomes or lysosomes resulting in cargo degradation. The formation of an autophagosome is driven by the temporally and spatially coordinated action of several major protein complexes of the core autophagy machinery: the ULK1 complex, the PIK3C3/VPS34 complex (class III phosphatidylinositol 3-kinase [PtdIns3K] complex), ATG9, ATG2, and the Atg8-family conjugation system [9]. The ULK1 complex, consisting of the serine/threonine kinase ULK1 (or its homolog ULK2), RB1CC1/FIP200, ATG13 and ATG101, is thought to be the most upstream autophagy-initiating complex that integrates cellular signals to drive autophagosome formation. In the absence of components of the ULK1 complex, the recruitment of downstream autophagy proteins is blocked and phagophore initiation is impaired [10–13]. In response to ULK1 complex assembly at the autophagosome formation site, the PtdIns3K complex, consisting of the lipid kinase PIK3C3/VPS34, ATG14, BECN1/Beclin-1, NRBF2 and PIK3R4/VPS15, is recruited to generate phosphatidylinositol-3-phosphate (PtdIns3P) on the emerging phagophore. PtdIns3P functions as a recruitment signal for downstream autophagy proteins, such as proteins of the WIPI family, which in turn lead to the recruitment of the Atg8-family protein conjugation machinery [9,10]. Ubiquitin-like proteins of the Atg8 family (GABARAP, GABARAPL1, GABARAPL2, LC3A, LC3B, LC3C) are conjugated to phosphatidylethanolamine (PE) in the expanding phagophore membrane, which is dependent on E1-like (ATG7), E2-like (ATG3) and E3-like (ATG12–ATG5-ATG16L1 complex) activity. Atg8-family conjugation is a hallmark of conventional autophagy. Many autophagy proteins contain LC3-interacting region (LIR) motifs that facilitate Atg8-family protein interactions. Thus, Atg8-family protein conjugation is important for the recruitment and stabilization of autophagy proteins on phagophores, fusion of autophagosomes with lysosomes and tethering of cargo to the phagophore [14,15]. In the absence of Atg8-family protein conjugation, autophagy is severely impaired but autophagosomes can still form, though at a reduced rate. However, autophagosomes fail to efficiently fuse with lysosomes [16] and show a defect in the degradation of the inner autophagosomal membrane [17].

In addition to the core autophagy machinery described above, selective forms of autophagy, such as mitophagy, require initial priming of cargo with “eat-me” signals that 1) recruit the core autophagy machinery to (locally) drive the formation of an autophagosome, and 2) mediate efficient tethering of the cargo to the expanding phagophore. Priming signals, such as cargo ubiquitination, can lead to the recruitment of receptors of the sequestosome-like receptor/SLR family, including SQSTM1/p62, CALCOCO2/NDP52, OPTN, TAX1BP1, and NBR1. These receptors can directly interact with RB1CC1 and thus recruit the ULK1 complex to locally drive the formation of an autophagosome around the cargo [18–27]. Likewise, many autophagy receptors contain LIR motifs for the engagement of Atg8-family proteins to firmly tether the cargo to the growing phagophore [15].

The selective autophagic degradation of mitochondria (termed mitophagy) serves as an important mitochondrial quality control pathway to eliminate dysfunctional mitochondria from cells and many diseases, in particular neurodegenerative disorders such as Parkinson disease (PD), are characterized by mitochondrial dysfunction [28–34]. Thus, enhancing mitophagy, such as via targeted organelle degradation, could be a novel therapeutic approach in PD. However, given that autophagy initiation requires multiple protein complexes, it is not yet clear which components are best suited to be mitochondrially recruited and drive efficient mitophagy. Therefore, the initial aim of this study was to elucidate the minimal requirements for mitophagy, with regards to identifying candidate autophagy proteins that, when recruited to mitochondria, resulted in their lysosomal delivery. Second, upon identifying such candidates, we aimed to use their recruitment to obtain further mechanistic insight into the process of mitophagy itself. We targeted different proteins of the core autophagy machinery to mitochondria by tagging them with an anti-mCherry or anti-GFP nanobody and expressing them in *mito*-QC (mCherry-GFP-FIS1[101-152]) reporter cells [35]. The cytosolically exposed outer mitochondrial membrane (OMM) mCherry-GFP tag of *mito*-QC allows the recruitment of the nanobody-tagged autophagy protein as well as functioning as a read-out for mitophagy, as visualized by the appearance of mCherry-only fluorescing mitolysosomes. With a focus on ULK1, ATG16L1 and the Atg8-family proteins, we found that the recruitment of each to mitochondria induced varying levels of mitophagy. Mitophagy was most potently induced when recruiting an RB1CC1- and WIPI2-binding peptide derived from ATG16L1 (ATG16L1[100-250]). Interestingly and in contrast to the other autophagy proteins analyzed here, the ATG16L1-derived peptide induced an unconventional mitophagy pathway that was still dependent on RB1CC1 and PIK3C3 activity but occurred independently of ATG5 and hence Atg8-family protein conjugation. Here, mitochondria were alternatively delivered to lysosomes via uptake into early endosomes.

## Results

### Targeting exogenous ULK1 to mitochondria induces mitophagy

**Figure 1.**
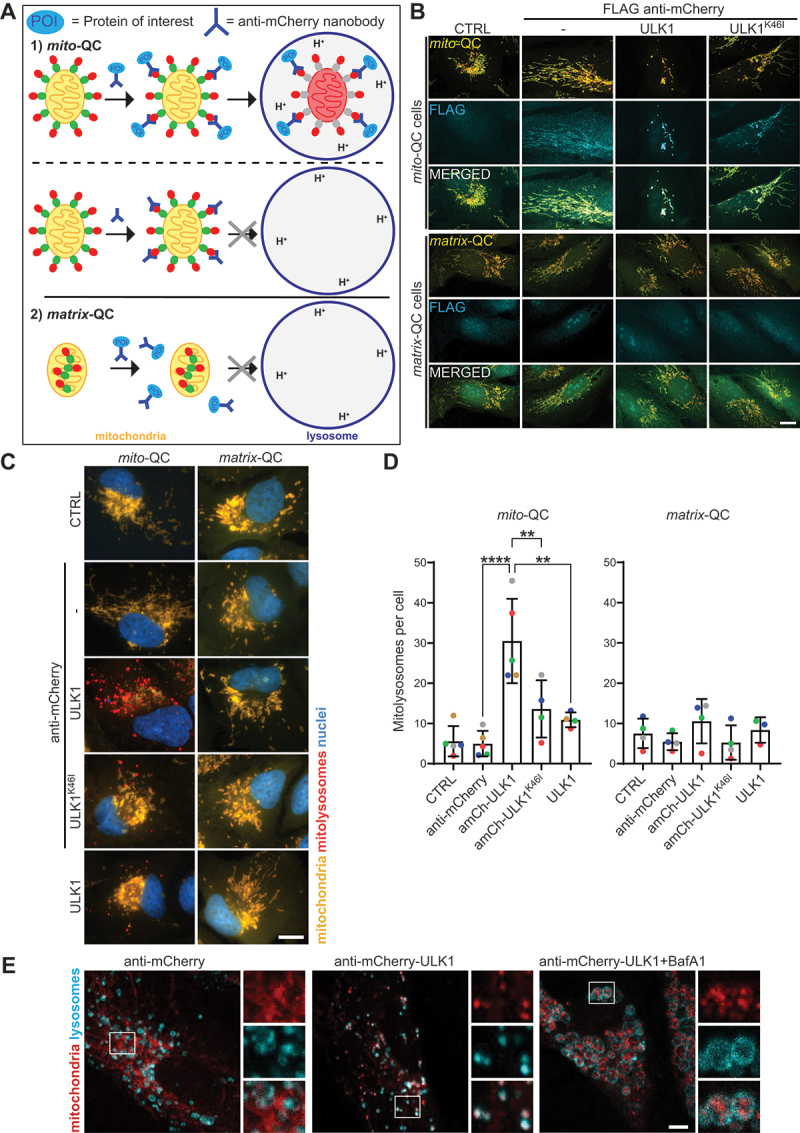
Targeting exogenous ULK1 to mitochondria induces mitophagy. (A) Experimental set-up. (1) The *mito*-QC reporter (mCherry-GFP-FIS1[101–152]) is localized to the outer mitochondrial membrane. Expression of a fusion product of the POI (protein of interest) conjugated to either an anti-GFP or an anti-mCherry nanobody allows the targeting of the POI to mitochondria. If the recruitment of the POI to mitochondria is sufficient to induce mitophagy, mitolysosomes are expected to form that can be detected by the acidic lysosomal quenching of the GFP fluorescence of the *mito*-QC reporter, whereas the mCherry fluorescence remains stable (mitolysosomes thus appear as mCherry-only puncta). The expression of the nanobody alone without fusion to the POI serves as a negative control. (2) The *matrix*-QC reporter (COX8[1–36]-COX8[1–36]-mCherry-GFP) is localized to the mitochondrial matrix. Hence, the mCherry-GFP tandem is not accessible to the cytosolic nanobody-POI construct and there is no mitochondrial localization of the POI and no mitolysosome formation. (B) ARPE-19 *mito*-QC or *matrix*-QC cells were transduced with the indicated proteins for 48 h, or were not transduced (CTRL), before immunofluorescence staining using an anti-FLAG antibody and analysis by confocal microscopy. Scale bar: 10 µm. (C and D) ARPE-19 *mito*-QC or *matrix*-QC cells were transduced with the indicated proteins for 48 h before widefield microscopy analysis. (C) Representative images. Scale bar: 10 µm. (D) Quantification of mitophagy showing the number of mitolysosomes per cell. Statistics: One-way ANOVA + Tukey’s multiple comparisons test. Left: *mito*-QC cells: shown is the mean number of mCherry-only mitolysosomes per cell from 4-5 independent experiments with a minimum of 49 cells being analyzed for each condition in each experiment. Right: *matrix*-QC cells: shown is the mean number of mCherry-only mitolysosomes per cell from 3-4 independent experiments with a minimum of 45 cells being analyzed for each condition in each experiment. (E) ARPE-19 cells stably expressing mitochondrially localized mCherry-FIS1[101-152] were transduced with the indicated proteins for 48 h and treated with bafilomycin A_1_ (BafA1, 50 nM) or DMSO (0.05%) for the last 24 h before immunofluorescence staining using an anti-LAMP1 antibody and confocal microscopy analysis. Scale bar: 5 µm.

Nanobodies have been used previously in the context of targeted protein degradation to target E3 ligases to a protein of interest (POI) and induce its proteasomal degradation [36,37]. Here, we aimed to use nanobodies in the context of targeted organelle degradation: to recruit a component of the autophagic machinery to mitochondria to induce their lysosomal degradation (i.e. mitophagy, see Figure 1A). To do this, we took advantage of the *mito*-QC reporter that consists of an mCherry-GFP tandem fused to the OMM targeting sequence derived from the protein FIS1 (mCherry-GFP-FIS1[101-152]) [35]. Thus, the mCherry-GFP tandem is localized at the cytosolic surface of mitochondria. The POI was fused with either an anti-GFP (abbreviation: aGFP) or an anti-mCherry (abbreviation: amCh) nanobody and the nanobody-POI construct was expressed in cells via transient retroviral transduction. Once expressed, the nanobody binds to the mCherry-GFP tandem on the surface of mitochondria resulting in the mitochondrial localization of the POI. If the POI subsequently triggers mitophagy, mitochondria will be delivered to lysosomes, where the GFP fluorescence of the *mito*-QC reporter is quenched, due to the acidic pH in lysosomes, and only the mCherry fluorescence remains. Hence, the level of mitophagy can be calculated by comparing the loss of mCherry-GFP mitochondrial signal (which overlaps as a yellow color), to the appearance of mCherry-only mitolysosome signal (red color) (Figure 1A). To control for this set of experiments, the nanobody without fusion to the POI was expressed in cells or, alternatively, the mCherry-GFP tag was targeted to the mitochondrial matrix instead of the mitochondrial surface, using a tandem matrix targeting sequence derived from COX8 (COX8[1–36]-COX8[1–36]-mCherry-GFP). For simplicity, we termed this reporter *matrix*-QC. The *matrix*-QC reporter is not accessible to the nanobody-POI construct and hence should not result in mitochondrial localization of the POI (Figure 1A). Importantly, the *mito*-QC and the *matrix*-QC reporters detect mitophagy (mCherry-only mitolysosomes) with a very similar sensitivity, as tested in response to two different chemical mitophagy inducers: deferiprone (DFP, an iron chelator [35]) or carbonyl cyanide m-chlorophenylhydrazone (CCCP, a proton ionophore [38]) (Figure S1A).

The first POI we targeted to mitochondria was ULK1, the kinase component of the autophagy-initiating ULK1 complex that translocates to mitochondria in response to mitophagic stimuli and mitochondrial priming [19,23,39]. ULK1, or a kinase-dead variant (K46I) [40], was fused (at its N terminus) to a FLAG-tagged anti-mCherry nanobody and expressed in our reporter cell lines (Figure S1B). In *mito*-QC cells, all the FLAG-tagged constructs localized to the *mito*-QC reporter, indicating mitochondrial recruitment (Figure 1B). Of note, staining with anti-FLAG requires permeabilization of fixed cells, which also results in the loss of lysosomal acidity, dequenching of lysosomal GFP and loss of mCherry-only mitolysosome staining (compare with non-permeabilized cells in Figure 1C). Mitochondrial localization was confirmed by co-staining with the mitochondrial protein COX4/COXIV (Fig. S1C). Importantly, no mitochondrial recruitment was seen in *matrix*-QC cells, demonstrating specificity of recruitment (Figure 1B). Interestingly, there was a change in the mitochondrial morphology away from the tubular network toward more condensed and aggregated mitochondria when recruiting exogenous ULK1 to mitochondria (Figure 1B; S1C). Next, mitophagy induction in response to the mitochondrial recruitment of ULK1 was investigated. Importantly here, cells were not permeabilized to preserve the acidic lysosomal pH and the ability to detect mCherry-only mitolysosomes. Expression of anti-mCherry-ULK1 in *mito*-QC cells for 48 h was sufficient to induce the formation of mCherry-only mitolysosomes (Figure 1C,D). All other constructs, including the nanobody alone, anti-mCherry-ULK1^K46I^ (kinase-dead), or ULK1 without the nanobody, failed to induce significant mitophagy. Likewise, expression of all constructs in *matrix*-QC cells failed to induce mitophagy (Figure 1C,D).

ULK1 normally forms a complex with RB1CC1, ATG13 and ATG101. To determine whether exogenous anti-mCherry-ULK1 also forms this complex, we immunoprecipitated (IP’d) anti-mCherry-ULK1, via its FLAG tag. Indeed, endogenous RB1CC1 and ATG13 were co-IP’d, demonstrating that the entire ULK1 complex is recruited to mitochondria alongside exogenous anti-mCherry-ULK1 (Figure S1D). Interestingly, the pool of ATG13 that co-IP’d with kinase active WT anti-mCherry-ULK1 showed a lower electrophoretic mobility than the pool of ATG13 that co-IP’d with kinase-dead anti-mCherry-ULK1^K46I^, suggesting that WT anti-mCherry-ULK1 is active and phosphorylates substrates, including ATG13 [41–44].

As an alternate measure of mitophagy, anti-mCherry-ULK1 was expressed in cells with mCherry-tagged mitochondria (mCherry-FIS1[101-152]) that were treated with/without bafilomycin A_1_ (BafA1) and co-stained with the lysosomal marker LAMP1 (Figure 1E). Co-localization of mCherry and LAMP1 was clearly observed when the mitochondrially targeted ULK1 was expressed. Addition of BafA1, which prevents lysosomal degradation leading to lysosomal swelling, allowed clear visualization of mCherry puncta within the lysosomal lumen.

We also tested the induction of mitophagy using an anti-GFP nanobody for targeting of ULK1 to mitochondria (Figure S1E-G). FLAG anti-GFP-ULK1 co-IP’d with the *mito*-QC reporter (Figure S1E), demonstrating that the anti-GFP nanobody can also be used for targeting a POI to *mito*-QC mitochondria. As with the anti-mCherry nanobody, expression of WT anti-GFP-ULK1, but not kinase-dead anti-GFP-ULK1^K46I^, was sufficient to induce mitophagy as assessed by microscopy analysis of mitolysosome formation (Figure S1F). As a reporter-independent method to monitor mitophagy, the loss of mitochondrial mass upon ULK1 targeting was measured biochemically via a CS (citrate synthase) assay (Figure S1G). Mitochondrial citrate synthase activity was reduced upon 72 h of anti-GFP-ULK1 expression in *mito*-QC cells compared to the expression of the anti-GFP nanobody alone (Figure S1G). Thus, these data are consistent with the fluorescence-based *mito*-QC assay in showing that the recruitment of exogenous ULK1 to mitochondria is sufficient to induce mitophagy. Taken together, these results suggests that the mitochondrial recruitment of catalytically active ULK1 is sufficient to induce mitophagy.

### Recruiting proteins of the Atg8-family protein conjugation system to mitochondria induces mitophagy

**Figure 2.**
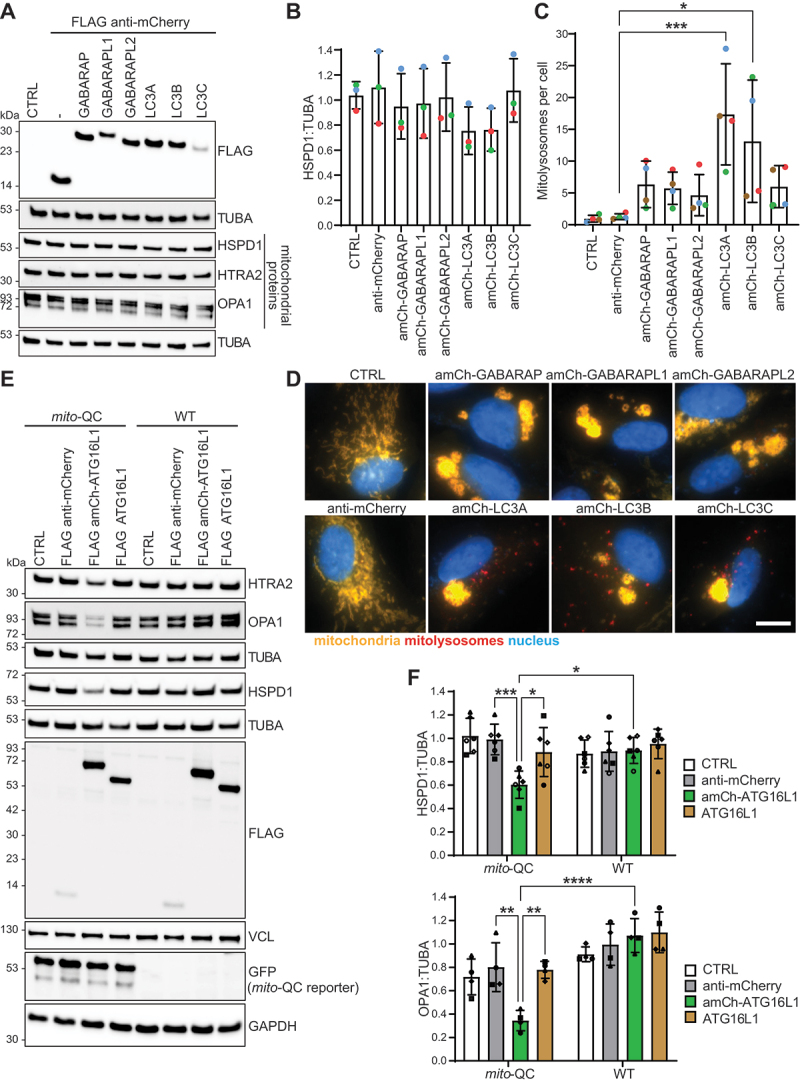
The mitochondrial recruitment of proteins of the Atg8-family protein conjugation system induces mitophagy. (A) ARPE-19 *mito*-QC cells were transduced (or not – CTRL) with the indicated proteins for 48 h and then lysed and processed for immunoblotting. (B) Quantification of mitochondrial HSPD1 levels from immunoblotting as in A, normalized to TUBA/tubulin from 3 independent experiments. Statistics: Two-way ANOVA + Dunnett’s multiple comparisons test (comparison of everything to anti-mCherry). (C) ARPE-19 *mito*-QC cells were transduced (or not – CTRL) with the indicated proteins for 48 h. Quantification of mitophagy from *mito*-QC assay showing the mean number of mitolysosomes per cell from 4 independent experiments with a minimum of 56 cells being analyzed for each condition in each experiment. Statistics: One-way ANOVA + Dunnett’s multiple comparisons test (comparison of everything to anti-mCherry). (D) Representative *mito*-QC images from cells transduced as in C. Scale bar: 10 µm. (E) ARPE-19 WT (no expression of the *mito*-QC reporter) or *mito*-QC cells were transduced with the indicated proteins (or were not transduced [CTRL]) for 48 h before cell lysis and immunoblot analysis. A Representative immunoblot is shown. (F) Quantification of mitophagy (from cells treated as in E) showing the levels of the mitochondrial proteins HSPD1 and OPA1 normalized to TUBA from a minimum of 4 independent experiments. Statistics: Two-way ANOVA + Tukey’s multiple comparisons test.

Having established that the mitochondrial recruitment of ULK1 is sufficient to induce mitophagy, we next investigated whether the recruitment of downstream core autophagy proteins could also induce mitophagy. Here, we focused on proteins of the Atg8-family protein conjugation system, as these have previously been linked to phagophore cargo incorporation via LIR-containing autophagy receptors [15]. The different human Atg8-family proteins (GABARAP, GABARAPL1, GABARAPL2, LC3A, LC3B, LC3C) were fused to the FLAG anti-mCherry nanobody (via the N terminus, with the Atg8-family protein C terminus facing the cytosol). With the exception of the anti-mCherry-LC3C construct, all Atg8-family proteins were expressed to similar levels (Figure 2A). We do not know why the LC3C construct appears less stable in our system. Regardless, all constructs co-localized with mCherry-tagged (mCherry-FIS1[101-152]) mitochondria (Figure S2). Strikingly, the expression of all the mitochondrially targeted Atg8-family constructs resulted in significant clustering of mitochondria (Figure 2D; S2). This could relate to the known membrane tethering and fusion capabilities of Atg8-family proteins [45–49]. This clustering did not prevent mitophagy, as the mitochondrial recruitment of the LC3 subfamily significantly induced mitolysosome formation (Figure 2C,D), with the exception of anti-mCherry-LC3C, which induced lower levels of mitophagy probably owing to its lower expression level. In contrast, and somewhat surprisingly given the perceived degree of redundancy between Atg8-family proteins, the recruitment of the GABARAP subfamily only moderately enhanced mitophagy levels. Regardless, the level of mitophagy induced by the recruitment of the different Atg8-family proteins is relatively low, as shown by the overall minimal loss of mitochondrial proteins (Figure 2A,B).

**Figure 3.**
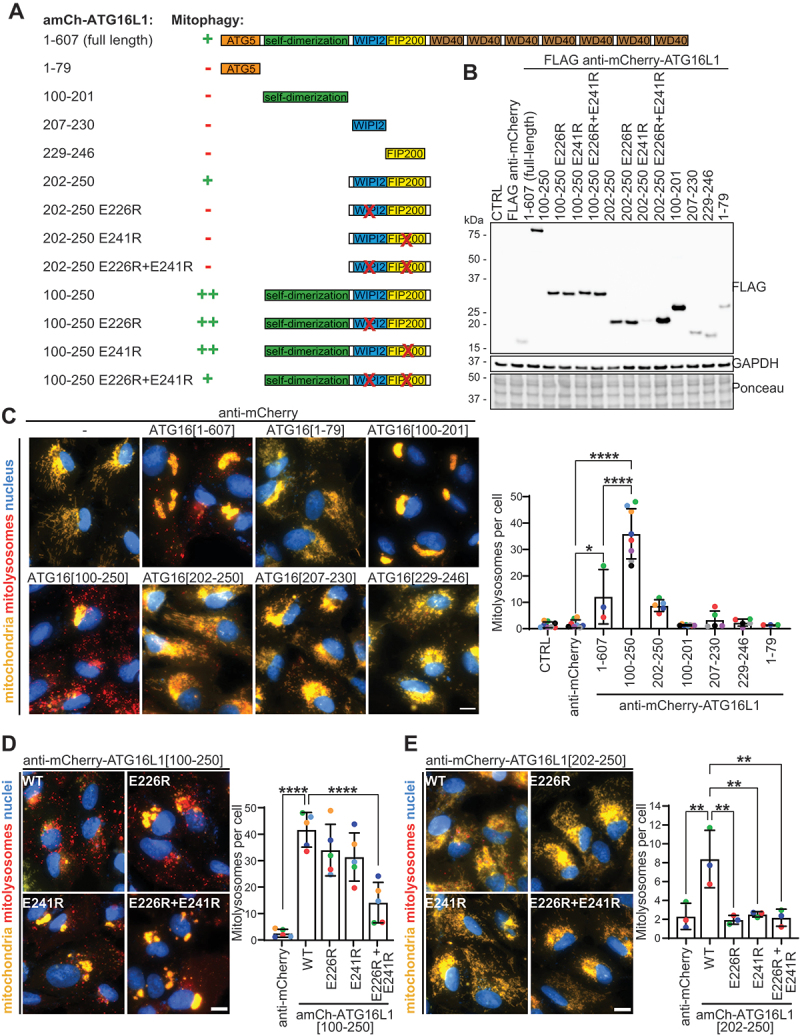
Recruiting RB1CC1- and WIPI2-binding ATG16L1[100-250] to mitochondria potently induces mitophagy. (A) Schematic of different ATG16L1-derived peptides. (B) ARPE-19 *mito*-QC cells were transduced with the indicated proteins (or not transduced [CTRL]) for 48 h, lysed and expression analyzed by immunoblot. (C, D and E) Representative widefield microscopy images of *mito*-QC assay in cells transduced to express the indicated proteins, as in B, for 48 h (scale bar: 10 µm). Quantification of mitophagy is shown on the right, indicating the mean number of mitolysosomes per cell from a minimum of 3 independent experiments with at least 57 cells analyzed per condition in each experiment. Statistics: One-way ANOVA + Tukey’s multiple comparisons test. Data shown in C-E were performed at the same time and utilize the same anti-mCherry controls.

Having established that the direct mitochondrial recruitment of Atg8-family proteins themselves is sufficient to induce mitophagy, we next investigated whether the mitochondrial recruitment of ATG16L1, as part of the Atg8-family protein-conjugating E3-like ATG12–ATG5-ATG16L1 complex, is also sufficient to induce mitophagy. Within the ATG12–ATG5-ATG16L1 complex, ATG16L1 determines the site of Atg8-family protein conjugation by interacting with WIPI2 [50] and ectopic localization of ATG16L1 to the plasma membrane induces Atg8-family protein conjugation there [51]. FLAG anti-mCherry-ATG16L1 co-localized with mCherry-tagged (mCherry-FIS1[101-152]) mitochondria and, as with the Atg8-family proteins, its expression caused a mitochondrial clustering phenotype. The mitochondrial clustering was dependent on mitochondrial targeting and was not observed by expressing FLAG-ATG16L1 (without the nanobody) (Fig.ure S2). The clustering phenotype is consistent with a report demonstrating that the ATG12–ATG5-ATG16L1 complex can tether giant unilamellar vesicles [52]. The mitochondrial recruitment of ATG16L1 induced significant mitophagy following 48 h of transduction, which was clearly indicated by the loss of mitochondrial proteins (OPA1, HSPD1/HSP60, or HTRA2/OMI) following immunoblot analysis of cell lysates (Figure 2E,F). This was also confirmed by the *mito*-QC assay (Figure 3C). The mitochondrial localization of ATG16L1 was essential for mitophagy induction, as the expression of anti-mCherry-ATG16L1 in WT cells (without expression of the *mito*-QC reporter) did not result in the loss of mitochondrial proteins (Figure 2E,F). Taken together, the mitochondrial recruitment of ATG16L1 or different Atg8-family proteins was sufficient to induce mitophagy.

## An RB1CC1- and WIPI2-binding peptide of ATG16L1, ATG16L1[100-250], potently induces mitophagy upon mitochondrial recruitment.

Given the strong induction of mitophagy by recruiting ATG16L1, we further investigated its mechanism of action. In a first step, we determined which region within ATG16L1 is responsible for driving mitophagy by creating and expressing several ATG16L1 truncation mutants fused to the anti-mCherry nanobody (Figure 3A,B). ATG16L1 is part of the E3-like ATG12–ATG5-ATG16L1 complex and contains an N-terminal domain (residues 1–79) for binding to ATG5 [53–55]; a coiled-coil region that encompasses a self-dimerization domain (residues 100–201) [53–55]; a WIPI2-binding region (residues 207–230) [50,56]; an RB1CC1-binding region (residues 229–246) [50,55,57,58]; and a C-terminal domain containing seven WD40 repeats [54]. The WIPI2- and RB1CC1-binding regions of ATG16L1 are essential for canonical starvation-induced autophagy [50,55,58,59]. Thereby, binding to WIPI2 is required to allow the recruitment of the ATG12–ATG5-ATG16L1 complex to phagophores and subsequent Atg8-family protein conjugation [50,60]. The C-terminal WD40 repeat domain of ATG16L1 is essential for conjugation of Atg8-family proteins to single membranes (CASM) during non-canonical autophagy, where Atg8-family proteins are conjugated to endolysosomal single membranes. This C-terminal section is however dispensable for canonical starvation-induced autophagy [59,61–63]. Therefore, we focused on the first section of ATG16L1 (residues 1–250), which is essential for canonical autophagy. Mitophagy induction in response to recruiting these different ATG16L1-derived peptides was assessed using the *mito*-QC assay (Figure 3A,C). As shown previously by immunoblotting (Figure 2E,F), the mitochondrial recruitment of full-length ATG16L1 induced a strong mitophagy response. In contrast, the mitochondrial recruitment of either the ATG5-binding (ATG16L1[1-79]) 34, the self-dimerization (ATG16L1[100-201]), the WIPI2-binding (ATG16L1[207-230]) or the RB1CC1-binding (ATG16L1[229-246]) region of ATG16L1 was not sufficient to induce mitophagy (Figure 3C). However, it is important to note that the ATG5-binding, the WIPI2-binding and the RB1CC1-binding constructs were expressed at a slightly lower level (Figure 3B). Interestingly, mitochondrial clustering was associated with the recruitment of the self-dimerization domain of ATG16L1 (ATG16L1[100-201]), as the mitochondrial recruitment of all ATG16L1-derived peptides containing this region displayed this phenotype. However, mitochondrial clustering alone was not sufficient to induce mitophagy. The minimal tested region within ATG16L1 that could induce mitophagy upon mitochondrial recruitment encompassed ATG16L1[202-250], which contains both the WIPI2- and the RB1CC1-binding regions (Figure 3C). However, mitophagy was much more potently induced when the ATG16L1 clustering-inducing self-dimerization domain was also included with the WIPI2- and RB1CC1-binding peptide (ATG16L1[100-250]) (Figure 3C). Indeed, some cells completely lost all GFP fluorescence and only mCherry-only mitolysosomes remained upon ATG16L1[100-250] recruitment (Figure 3C). In summary, these results suggest that ATG16L1 drives mitophagy by recruiting the upstream RB1CC1 and WIPI2 autophagy machinery. To further confirm this, we next introduced point mutations that disrupt either WIPI2 (E226R mutation) or RB1CC1 (E241R mutation) binding [50] into mitophagy-inducing anti-mCherry-ATG16L1[100-250] (Figure 3D) or anti-mCherry-ATG16L1[202-250] (Figure 3E) constructs. Mitophagy induction was significantly impaired when both point mutations were present, indicating that direct binding to RB1CC1 and WIPI2 is essential for driving mitophagy in response to the mitochondrial recruitment of ATG16L1.

It is possible that the recruitment of the nanobody-POI to mitochondria results in mitochondrial depolarization, and it is this depolarization that drives mitophagy rather than the recruitment of the specific POI. To rule this out we stained mitochondria with the membrane-potential-sensitive dye MitoTracker Deep Red (Figure S3). Compared to cells treated with the depolarizing agent CCCP, the mitochondrial recruitment of ULK1, full-length ATG16L1, or ATG16L1[100-250] did not reduce MitoTracker staining. This supports our other data in that the mitochondrial recruitment of ULK1 or ATG16L1 is the driver of mitophagy.

### Mitophagy induction upon mitochondrial recruitment of ATG16L1[100-250] or ULK1 requires RB1CC1

**Figure 4.**
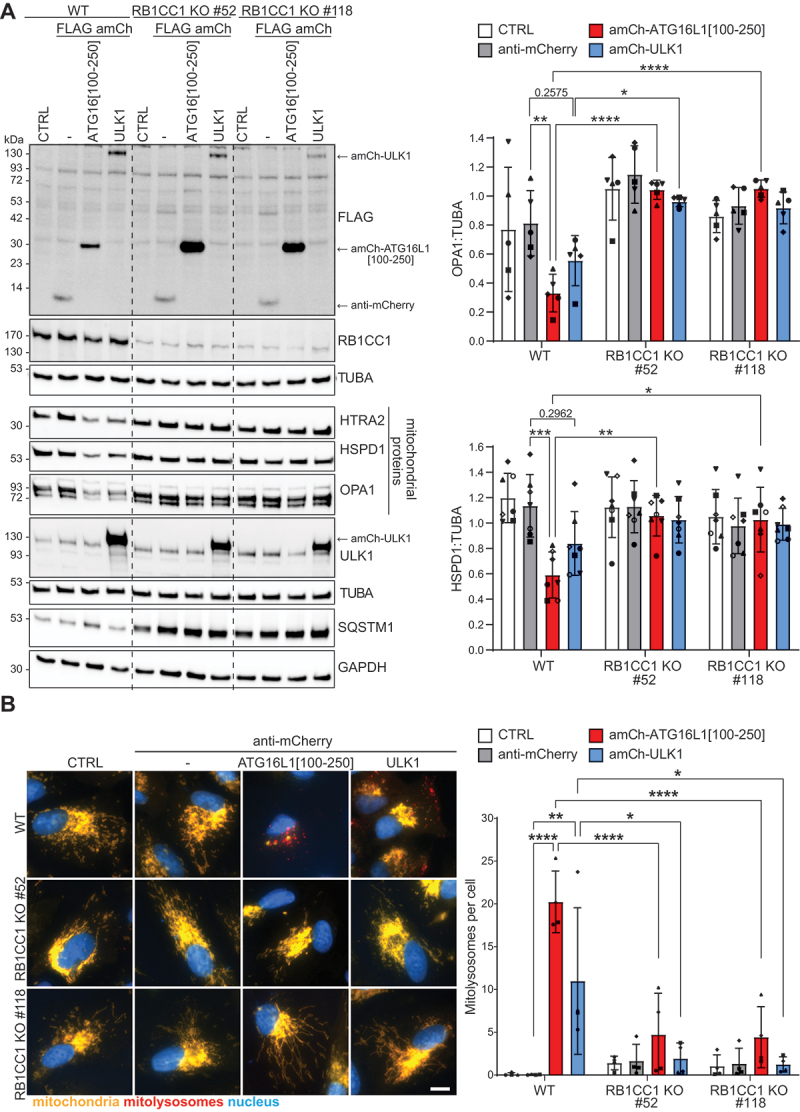
Mitophagy induction in response to the mitochondrial recruitment of ATG16L1[100-250] or ULK1 is blocked in *RB1CC1* KO cells. (A) WT, *RB1CC1* KO #52 or *RB1CC1* KO #118 ARPE-19 *mito*-QC cells were transduced to express the indicated proteins or not transduced (CTRL) for 48 h before cell lysis and immunoblot analysis. Left: Representative immunoblot. Right: Quantification of mitophagy showing the levels of the mitochondrial proteins HSPD1 and OPA1 normalized to TUBA from a minimum of 5 independent experiments. Statistics: Two-way ANOVA + Tukey’s multiple comparisons test. (B) WT, *RB1CC1* KO #52 or *RB1CC1* KO #118 ARPE-19 *mito*-QC cells were transduced to express the indicated proteins (or not transduced [CTRL]) for 48 h before microscopy analysis. Left: Representative images. Scale bar: 10 µm. Right: Quantification of mitophagy showing the mean number of mitolysosomes per cell from 4 independent experiments with a minimum of 30 cells being analyzed per condition in each experiment. Statistics: Two-way ANOVA + Tukey’s multiple comparisons test.

To validate the mitophagy pathways induced by the recruitment of ULK1 or ATG16L1 (and in particular the potent ATG16L1[100-250] peptide), we next investigated the requirement for the three major protein complexes regulating autophagy initiation: the ULK1 complex, the PIK3C3 complex, and the ATG12–ATG5-ATG16L1 complex. To examine the requirement for the ULK1 complex, the most upstream element of the core autophagy machinery, we generated *RB1CC1* knockout (KO) cells using CRISPR. RB1CC1 is essential for ULK1 complex activity and autophagosome formation [41,42,64]. Using guides targeting exon 7 of RB1CC1, we generated 2 distinct *RB1CC1* KO clones (#52 and #118) with deletions or insertions resulting in a frameshift and premature stop codons in both alleles for both clones (Figure S4). We do note that by immunoblotting (Figure 4A), a faint band of similar size to RB1CC1 is observed in both KO clones. However, we think this likely a nonspecific band given that sequencing could not detect a WT allele. Consistent with a block in autophagy upon loss of RB1CC1, SQSTM1/p62 accumulated in the two distinct *RB1CC1* KO clones (Figure 4A). Mitophagy induction in response to the mitochondrial recruitment of ATG16L1[100-250] was blocked in both *RB1CC1* KO clones, as assessed by immunoblotting for mitochondrial proteins (OPA1, HSPD1, HTRA2) (Figure 4A) or microscopy analysis of mitolysosome formation (Figure 4B). Thus, RB1CC1 is essential for mitophagy induction, consistent with the finding above that direct binding of ATG16L1[100-250] to RB1CC1 is required (Figure 3D,E). Furthermore, FLAG anti-mCherry-ATG16L1[100-250] accumulates in the *RB1CC1* KO clones (Figure 4A), indicating that it is degraded alongside mitochondria in WT cells but not in the *RB1CC1* KO cells.

Mitophagy induction following the mitochondrial recruitment of ULK1 was also blocked in *RB1CC1* KO cells (Figure 4). This is consistent with the requirement for the entire ULK1 complex for autophagy and the fact that anti-mCherry-ULK1 interacts with both RB1CC1 and ATG13 (Figure S1D). Noteworthily, the levels of FLAG anti-mCherry-ULK1 do not accumulate in the *RB1CC1* KO clones (Figure 4A), potentially owing to the lower level of mitophagy observed with this construct compared to the anti-mCherry-ATG16L1[100-250] construct (Figure 4). In summary, RB1CC1 is essential for mitophagy induction in response to the mitochondrial recruitment of either ATG16L1[100-250] or ULK1.

### Mitophagy induction upon mitochondrial recruitment of ATG16L1[100-250] or ULK1 requires PIK3C3/VPS34 activity

**Figure 5.**
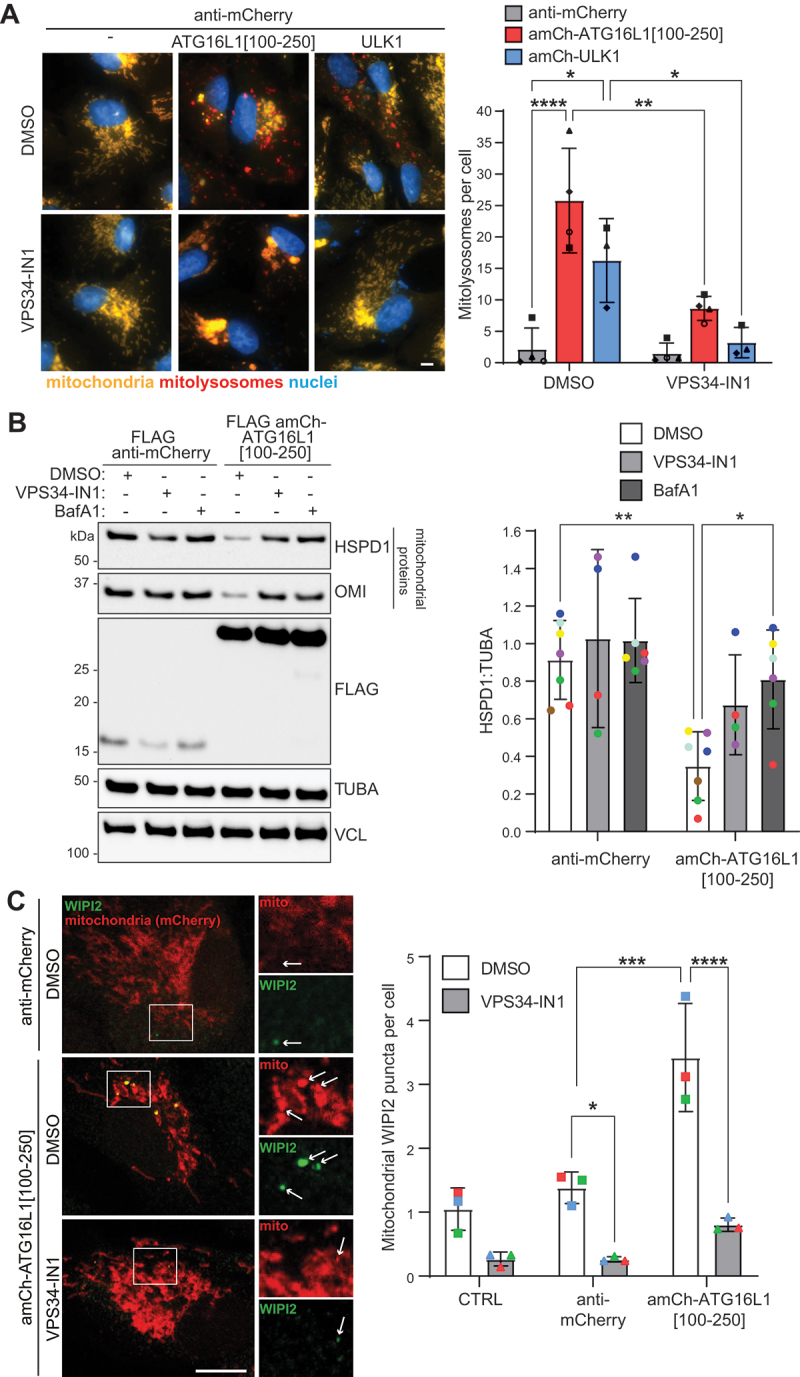
Mitophagy induction in response to the mitochondrial recruitment of ATG16L1[100-250] or ULK1 is dependent on PIK3C3 activity. (A) ARPE-19 *mito*-QC cells were transduced to express the indicated proteins for 48 h in the presence of VPS34-IN1 (1 μM) or DMSO (0.01%) for the entire 48 h. Cells were analyzed using widefield microscopy. Left: Representative images. Scale bar: 5 µm. Right: Quantification of mitophagy showing the mean number of mitolysosomes per cell from at least 3 independent experiments with a minimum of 33 cells being analyzed per condition in each experiment. Statistics: Two-way ANOVA + Tukey’s multiple comparisons test. (B) ARPE-19 cells stably expressing mitochondrially localized mCherry-FIS1[101-152] were transduced with the indicated proteins for 48 h in the presence of VPS34-IN1 (1 μM) for the entire 48 h or BafA1 (50 nM) for the last 24 h or were treated with DMSO as a control. Cells were lysed and samples were analyzed by immunoblotting. Left: Representative immunoblot. Right: Quantification of mitophagy showing the levels of the mitochondrial protein HSPD1 normalized to VCL from 4 independent experiments. Statistics: Two-way ANOVA + Tukey’s multiple comparisons test. (C) ARPE-19 cells stably expressing mitochondrially localized mCherry-FIS1[101-152] were transduced with the indicated proteins or were not transduced (CTRL) for 24 h in the presence of VPS34-IN1 (1 μM) or DMSO (0.01%) for the entire 24 h before immunofluorescence staining using an anti-WIPI2 antibody and confocal microscopy analysis. Left: Representative images. Scale bar: 10 µm. Right: Quantification of the mean number of WIPI2 puncta on mCherry-positive mitochondria from 3 independent experiments with a minimum of 34 cells being analyzed per condition in each experimental replicate. Statistics: wo-way ANOVA + Tukey’s multiple comparisons test.

Next, we investigated the requirement for the PtdIns3K complex for mitophagy induction by using the PIK3C3 inhibitor VPS34-IN1 [65]. Mitophagy induction by the recruitment of ATG16L1[100-250] or ULK1 was impaired in the presence of VPS34-IN1 (Figure 5A,B). In fact, the VPS34-IN1-driven block in mitophagy in response to the mitochondrial recruitment of ATG16L1[100-250] was similar to BafA1 treatment (Figure 5B). Hence, PIK3C3 activity is essential for mitophagy induction in this context. This latter experiment highlights the significant level of mitophagy caused by the ATG16L1[100-250] peptide, with over 70% of the mitochondrial markers being lost in a PIK3C3- and lysosomal-dependent manner.

WIPI2 puncta formation is often used as an early phagophore marker as WIPI2 is recruited in response to PtdIns3P generation by PIK3C3. WIPI2 in turn recruits the ATG12–ATG5-ATG16L1 complex for Atg8-family protein conjugation [50,66]. Discrete WIPI2 puncta were formed on mitochondria in response to the mitochondrial recruitment of ATG16L1[100-250] (Figure 5C) or ULK1 (Figure S5). Hence, early phagophore structures likely form around mitochondria in response to the mitochondrial recruitment of these constructs. Importantly, mitochondrial WIPI2 puncta formation in response to the recruitment of ATG16L1[100-250] was abrogated in the presence of VPS34-IN1 (Figure 5C). This indicates that WIPI2 puncta formation in response to ATG16L1[100-250] recruitment is not just a result of the direct interaction of ATG16L1[100-250] with WIPI2.

### Mitophagy induction by ATG16L1[100-250] and ULK1 have differing requirements for ATG5 and Atg8-family protein conjugation

**Figure 6.**
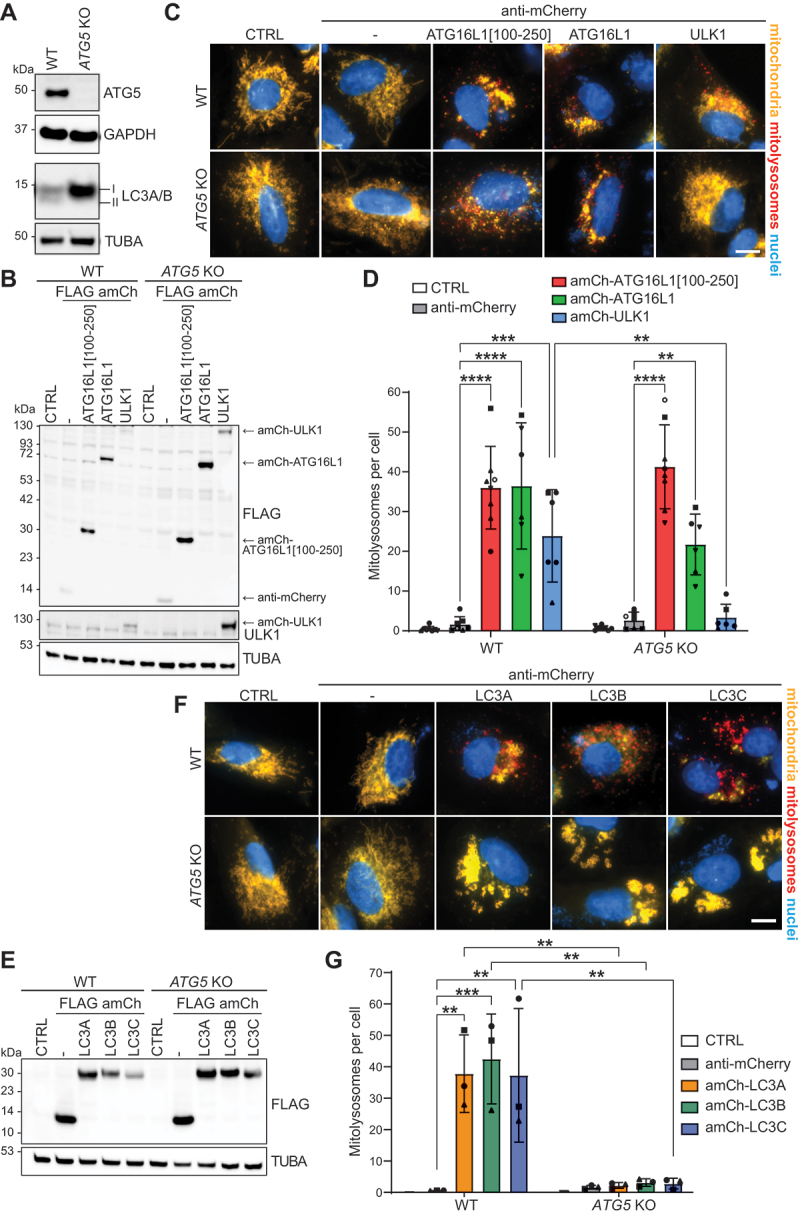
Mitophagy induction in response to the mitochondrial recruitment of ATG16L1[100-250] specifically does not require ATG5. (A) Immunoblot of WT and *ATG5* KO A549 cells. (B) WT or *ATG5* KO A549 *mito*-QC cells were transduced to express the indicated proteins, or were not transduced (CTRL), for 48 h before cell lysis. Shown is a representative immunoblot. (**C**) WT or *ATG5* KO A549 *mito*-QC cells were transduced as in B before widefield microscopy analysis. Representative *mito*-QC images are shown. Scale bar: 10 µm. (D) Quantification of mitophagy (in cells treated as in C) showing the mean number of mitolysosomes per cell from at least 6 independent experiments with a minimum of 40 cells per condition in each experiment. Statistics: Two-way ANOVA + Tukey’s multiple comparisons test. (E) WT or *ATG5* KO *mito*-QC A549 cells were transduced to express the indicated nanobody-LC3 proteins or were not transduced (CTRL) for 48 h before cell lysis. Shown is a representative immunoblot of expression levels. (F) WT or *ATG5* KO *mito*-QC A549 cells were transduced as in E for 48 h before widefield microscopy analysis. Representative images are shown. Scale bar: 10 µm. (G) Quantification of mitophagy (in cells treated as in F) showing the mean number of mitolysosomes per cell from 3 independent experiments with a minimum of 40 cells being analyzed per condition in each experiment. Statistics: Two-way ANOVA + Tukey’s multiple comparisons test.

Finally, the requirement for Atg8-family protein conjugation in mitophagy induction following the recruitment of ATG16L1[100-250] or ULK1 was investigated. For this, *ATG5* KO A549 cells (Figure 6; Figure S6A) or *atg5* KO MEFs (Figure S6B-D) were used to block Atg8-family protein lipid conjugation. The absence of ATG5 results in the loss of the high mobility LC3-II form on immunoblots and the accumulation of the unlipidated LC3-I form (Figure 6A; Figure S6B). As expected, mitophagy induction in response to the mitochondrial recruitment of ULK1 was completely blocked in *ATG5* KO cells (Figure 6B–D; S6B-D). However, unexpectedly, mitolysosome formation in response to the recruitment of either ATG16L1[100-250] or full-length ATG16L1 was not blocked in *ATG5* KO cells (Figure 6B–D; S6B-D). In the case of the anti-mCherry-ATG16L1[100-250] construct, mitophagy occurred as efficiently in *ATG5* KO cells as in WT cells (Figure 6D; S6D). In addition to the *mito*-QC assay, immunoblotting of mitochondrial proteins (OPA1, HSPD1) confirmed that mitophagy induction was unimpaired in *ATG5* KO cells following the mitochondrial recruitment of ATG16L1[100-250] (Figure S6A). Hence, although ATG16L1 is part of the Atg8-family protein conjugating ATG12–ATG5-ATG16L1 complex, the mitochondrial recruitment of ATG16L1[100-250] (which does not bind to ATG12–ATG5) or full-length ATG16L1 (which does form a complex with ATG12–ATG5) surprisingly induced an Atg8-family protein conjugation-independent mitophagy pathway.

Although mitophagy was not blocked when recruiting ATG16L1[100-250] or full-length ATG16L1 to mitochondria, their levels accumulated slightly in *ATG5* KO cells (Figure 6B). This could indicate that in WT cells, conventional and unconventional mitophagy pathways are induced, whereas in *ATG5* KO cells only the unconventional pathway can proceed but not the conventional one. This would explain the partial (but not significant) reduction of mitophagy observed when recruiting full-length ATG16L1. However, cellular clonal variation could be at play, and it is possible that the *ATG5* KO cells are more efficiently transduced with the construct-encoding retrovirus than the WT cells. This is conceivable given that the level of the FLAG anti-mCherry nanobody alone (which does not induce mitophagy) also slightly accumulates in the *ATG5* KO cells (Figure 6B).

Given that ATG16L1 could induce Atg8-family protein conjugation-independent mitophagy, we also tested the ATG5 requirement for mitophagy induced by the recruitment of the LC3 proteins themselves. As with the recruitment of ULK1, mitolysosome formation in response to the mitochondrial recruitment of LC3A, LC3B or LC3C was completely blocked in *ATG5* KO cells (Figure 6E–G). This demonstrates that the direct mitochondrial recruitment of Atg8-family proteins, using the nanobody system, is not sufficient to bypass the general requirement for Atg8-family protein conjugation.

To further corroborate these unexpected findings, we next tested the requirement for endogenous ATG16L1 in our mitophagy induction assays, as another component of the Atg8-family proteins conjugating ATG12–ATG5-ATG16L1 complex. This was also of particular interest considering that the presence of the self-dimerization domain (which allows the dimerization of ATG16L1[100-250] with endogenous ATG16L1) resulted in more potent mitophagy induction compared to the ATG16L1-derived peptide with just the RB1CC1- and WIPI2-binding regions (Figure 3A–C). As expected, Atg8-family protein conjugation was blocked in *atg16l1* KO MEFs but was rescued upon re-introduction of 3×HA-ATG16L1 or upon expression of anti-mCherry-ATG16L1. On the contrary, expression of anti-mCherry-ATG16L1[100-250] did not rescue Atg8-family protein conjugation (Figure S6E). Regardless of Atg8-family protein conjugation, mitolysosomes formed as efficiently in *atg16l1* KO MEFs as in the 3×HA-ATG16L1-rescued *atg16l1* KO MEFs in response to the mitochondrial recruitment of either ATG16L1[100-250] or full-length ATG16L1 (Figure S6F). Thus, endogenous ATG16L1 is dispensable for mitophagy induction in this context.

In summary, we found that depending on which autophagy proteins are recruited to mitochondria, mechanistically different mitophagy pathways can be induced. Whereas the mitochondrial recruitment of ULK1 or LC3 proteins induces a conventional ATG5-dependent mitophagy pathway, the mitochondrial recruitment specifically of ATG16L1 primes mitochondria for an unconventional ATG5-independent mitophagy pathway.

### Mitochondria are taken up into early endosomes in response to the mitochondrial recruitment of ATG16L1[100-250]

**Figure 7.**
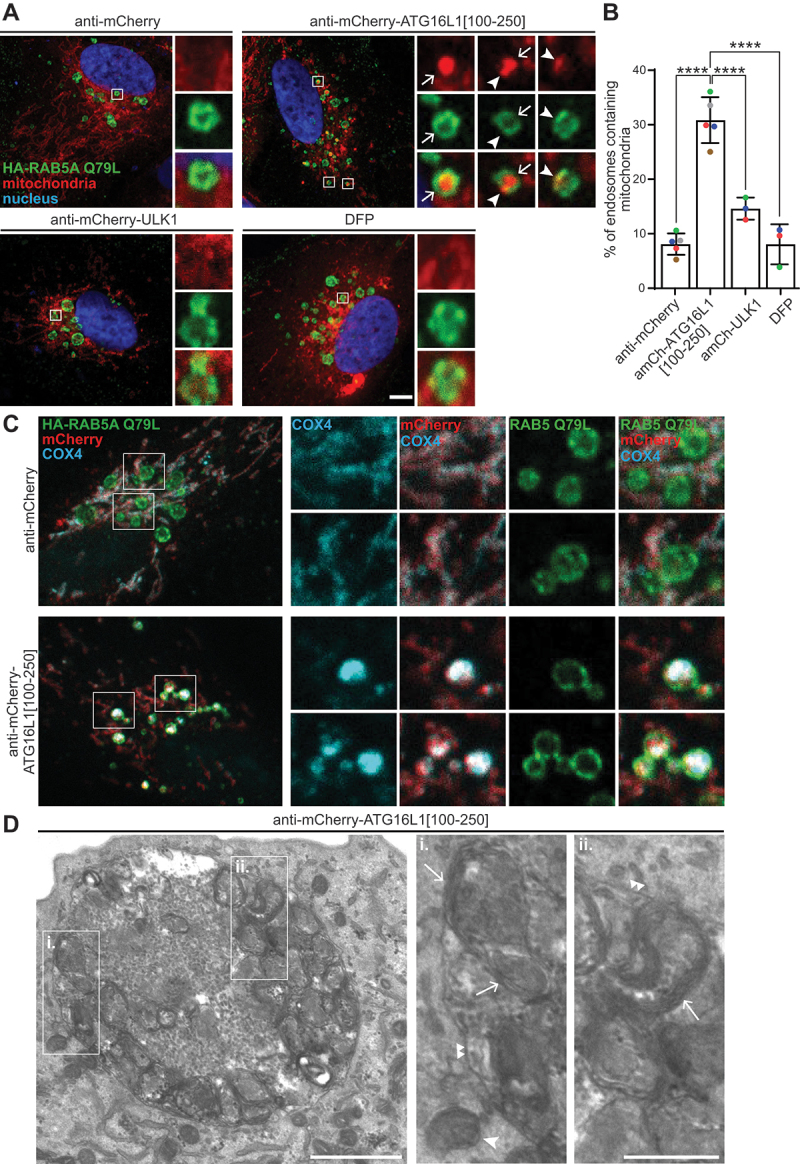
Mitochondria are taken up into early endosomes in response to the mitochondrial recruitment of ATG16L1[100-250]. (A) ARPE-19 cells stably expressing HA-RAB5A^Q79L^ and mitochondrially localized mCherry-FIS1[101-152] were transduced to express the indicated proteins, or were treated with DFP (1 mM), for 48 h before immunofluorescence staining with anti-HA and confocal microscopy analysis. Representative images are shown. Scale bar: 5 µm. Arrows: mitochondrial material inside of early endosomes. Arrowheads: mitochondrial material at the edge of early endosomes (possibly mitochondria in the process of being taken up). (B) Quantification of the percentage of endosomes containing mitochondrial material from at least 3 independent experiments with a minimum of 10 cells being analyzed per condition in each experiment. Statistics: One-way ANOVA + Tukey’s multiple comparisons test. (C) ARPE-19 cells, transduced as in A, were stained using antibodies against HA and the endogenous mitochondrial protein COX4. Shown are representative confocal images. Scale bar: 5 µm. (D) ARPE-19 cells, as in A, were processed for transmission electron microscopy analysis. Shown is a representative image of an enlarged early endosome in anti-mCherry-ATG16L1[100-250] treated cells. See also Fig. S7C. Scale bar: 1 µm, or 0.5 µm for insets i. And ii. Arrow: mitochondrial structures inside of early endosomes; large arrowhead: mitochondria close to early endosomes; small double arrowheads: limiting single endosomal membrane.

Atg8-family proteins are important for the efficient formation of autophagosomes and for fusion of autophagosomes with lysosomes [16,17]. As Atg8-family protein conjugation was completely dispensable for mitophagy induction in response to the mitochondrial recruitment of ATG16L1[100-250], mitochondria are likely delivered to lysosomes in an unconventional way. Apart from the autophagic route, material destined for lysosomal degradation can also be delivered to lysosomes via endosomes. Indeed, an alternative microautophagy-like pathway of mitochondrial degradation was described previously during which mitochondria are delivered to lysosomes independently of ATG5 by sequestration into early endosomes in response to FCCP treatment in PRKN/parkin-expressing cells [67]. In order to investigate a possible similar mechanism of lysosomal delivery in our system, we stably expressed a GTPase-deficient and constitutively active form of RAB5 (HA-RAB5A^Q79L^) in ARPE-19 cells to drive the formation of hyperfused enlarged early endosomes [68]. This facilitates visualization of endosomal contents under the microscope. In response to the mitochondrial recruitment of ATG16L1[100-250] specifically, mitochondrial material was frequently observed inside, or at the edge (possibly mitochondria in the process of being taken up) of, enlarged early endosomes (Figure 7A,B). Importantly, the uptake of mitochondria into enlarged early endosomes was specific to the ATG16L1[100-250]-driven mitophagy pathway. The iron chelator DFP is one of the most potent chemical inducers of conventional (ATG5-dependent) mitophagy [35] and treatment of cells with DFP induces similar levels of mitophagy as compared to the mitochondrial recruitment of ATG16L1[100-250] (Figure S7A,B). However, in contrast to ATG16L1[100-250] recruitment, DFP treatment, or the mitochondrial recruitment of ULK1, failed to cause an accumulation of mitochondrial content within the enlarged early endosomes (Figure 7A,B). Alongside the mitochondrial mCherry-FIS1[101-152] reporter, the endogenous inner mitochondrial membrane protein COX4 was also found in enlarged early endosomes upon mitochondrial recruitment of ATG16L1[100-250] (Figure 7C). Moreover, using transmission electron microscopy with the ATG16L1[100-250] recruitment, we observed very electron-dense structures that resemble mitochondrial fragments inside of, or in close vicinity to, enlarged early endosomes (Figure 7D). In contrast, while the enlarged early endosomes of control (anti-mCherry only) or anti-mCherry-ULK1 expressing cells contained intracellular material, they appeared to lack the characteristic mitochondrial fragments seen upon expression of anti-mCherry-ATG16L1[100-250] (Figure S7C). This implies that ATG5-dependent mitophagy involves fusion of mitophagosomes with endocytic compartments.

### Targeting of ATG16L1[100-250] to peroxisomes induces pexophagy

Considering that the recruitment of ATG16L1[100-250] to mitochondria was a potent mitophagy inducer, we next investigated whether its recruitment to a different cargo could also induce its lysosomal turnover. Peroxisomes are important organelles that can be autophagocytosed in response to the same signals as mitochondria [69]. To investigate whether the targeting of ATG16L1[100-250] to peroxisomes is sufficient to induce pexophagy (autophagy of peroxisomes), we generated cells expressing an mCherry-GFP tandem reporter on the outer surface of peroxisomes (mCherry-GFP-PEX26[245-274]) [70,71] or we used cells expressing an mCherry-GFP tandem reporter that is localized in the lumen of peroxisomes (mCherry-GFP-SKL) [69]. Therefore, as with the *mito*-QC vs *matrix*-QC (Figure 1), pexophagy should only be induced with the surface-localized reporter where the nanobody-POI construct can be recruited (Figure S7D). This proved to be the case as the expression of anti-mCherry-ATG16L1[100-250] in cells containing the surface-localized mCherry-GFP-PEX26[245-274] resulted in the formation of mCherry-only pexolysosomes, whereas the expression of anti-mCherry-ATG16L1[100-250] in cells containing lumen-localized mCherry-GFP-SKL did not (Figure S7E and F). Moreover, pexolysosome formation in response to the peroxisomal recruitment of ATG16L1[100-250] was sensitive to BafA1 treatment (Figure S7F). Hence, the recruitment of ATG16L1[100-250] to peroxisomes is sufficient to induce pexophagy and suggests that ATG16L1[100-250] recruitment can likely function as a universal signal for lysosomal organelle turnover.

**Figure 8.**
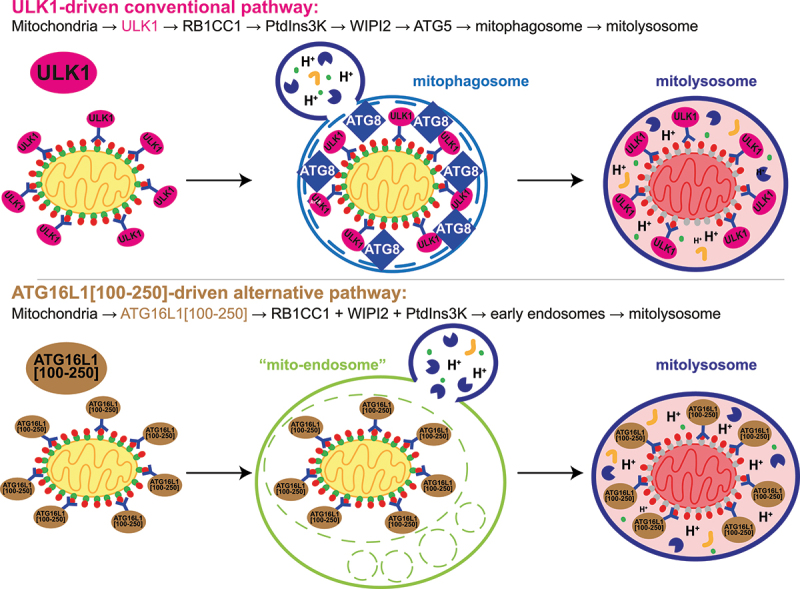
The recruitment of different autophagy proteins to mitochondria induces distinct mitophagy pathways. Model of how the mitochondrial recruitment of ULK1 versus ATG16L1[100-250] may drive different mitophagy pathways. ATG8: mammalian Atg8-family protein.

In conclusion, we found that the mitochondrial recruitment of ULK1 induces a conventional mitophagy pathway that requires RB1CC1, PIK3C3 activity and Atg8-family protein conjugation (Figure 8). On the other hand, the mitochondrial recruitment of an RB1CC1- and WIPI2-binding peptide derived from ATG16L1 (ATG16L1[100-250]) was capable of inducing a novel unconventional mitophagy pathway that, while still requiring RB1CC1 and PIK3C3 activity, occurs independently of Atg8-family protein conjugation. In this latter scenario, mitochondria are delivered to lysosomes via uptake into early endosomes (Figure 8).

## Discussion

### Hierarchy of core autophagy complexes

In this study we aimed to identify proteins that when targeted to mitochondria could induce mitophagy. Not only would this help validate candidate proteins for the future development of targeted organelle degradation therapies but also provide mechanistic insight into the process of mitophagy. We analyzed the recruitment of proteins related to the main core autophagy-induction machinery, namely ULK1, ATG16L1 and the different Atg8-family proteins. The classical hierarchical model of autophagy suggests that ULK1 complex activation leads to activation and recruitment of the PtdIns3K complex, which in turn leads to the recruitment of the ATG12–ATG5-ATG16L1 complex and concomitant Atg8-family protein lipidation. Perhaps unsurprisingly, our data shows that this simple hierarchy is much more complex as mitophagy in response to the recruitment of downstream ATG16L1 still required the upstream ULK1 complex and PIK3C3 activity to induce mitophagy. Moreover, mitophagy in response to the mitochondrial recruitment of Atg8-family proteins themselves still required ATG5 and thus Atg8-family protein conjugation. In the case of the Atg8-family proteins, it is possible that they directly recruit the upstream autophagy machinery as it is well established that many core autophagy proteins contain Atg8-family protein-interacting LIR motifs [72–77]. Alternatively, the anti-mCherry-Atg8-family protein constructs might recruit existing forming phagophores and tether them around mitochondria.

While all autophagy proteins tested here induced mitophagy to some level compared to control cells, the amount of mitophagy was dependent on the identity of the core component recruited. While ATG16L1, and peptides derived from this protein (namely the 100–250 peptide), induced strong levels of mitophagy, recruitment of the kinase-active upstream ULK1 protein was less potent but still a capable mitophagy inducer. Likewise, the mitochondrial recruitment of the LC3 family of proteins robustly induced mitophagy. Interestingly, mitophagy levels were higher compared to the recruitment of GABARAP family proteins. While we cannot yet explain the different levels of mitophagy induced by the different components, this does hint that the level of mitophagy induced could be fine-tuned for any therapeutic advantage by recruiting different components. However, it is important to note that the method of recruitment, via the nanobody-mCherry/GFP system, could impose steric restrictions that limit the mitophagy-inducing efficiency of some of the proteins.

### Unconventional mitophagy pathways

One of the most interesting and unexpected aspects of this study was that the recruitment of ATG16L1 to mitochondria induced a non-canonical pathway of mitophagy that bypassed the need for ATG5 and hence Atg8-family protein conjugation. Here, mitochondria were alternatively delivered to lysosomes via uptake into early endosomes. This alternate pathway could perhaps help explain the potent level of mitophagy induced by the ATG16L1[100-250] peptide. Importantly, the induction of this unconventional mitophagy pathway still required the upstream core autophagy machinery. Firstly, mitophagy induction was dependent on a direct interaction of ATG16L1 with RB1CC1 and WIPI2 (ATG16L1[100-250]). This is consistent with an earlier report showing that mitophagy was induced when recruiting the ATG16L1[100-250] peptide to mitochondria using a chemically inducible targeting system in HeLa cells, though in this report mitophagy induction was only related to binding to RB1CC1 and assumed to proceed in a conventional manner [23]. Secondly, apart from binding to RB1CC1 and WIPI2, we found that mitophagy induction in response to ATG16L1[100-250] also required PIK3C3 activity. While PIK3C3 activity is also needed for endosomal function [78], we found that WIPI2 puncta formed on mitochondria in response to mitochondrial ATG16L1[100-250] recruitment and this was dependent on PIK3C3 activity, thus arguing for a role for the autophagic PtdIns3K complex I rather than the endosomal PtdIns3K complex II. Taken together, this means the same upstream autophagy machinery (ULK1 complex, PIK3C3 activity, WIPI2 puncta formation) is required/involved for the unconventional mitophagy pathway, as it is for the more conventional pathways induced upon ULK1 or LC3 recruitment. However, these mitophagy pathways diverge at the level of ATG5.

Interestingly, ATG5-independent mitochondrial uptake into early endosomes was reported previously in response to a high dose treatment of FCCP in PRKN-overexpressing MEFs, as well as in neonatal cardiomyocytes in response to simulated ischemia/reperfusion [67]. However, here mitochondria were thought to be directly sequestered into early endosomes via the ESCRT machinery, in an ULK1/2 complex-independent manner. In contrast, we found that the ATG16L1[100-250]-driven mitophagy pathway was dependent on the presence of RB1CC1 and involved the PIK3C3-dependent formation of WIPI2 puncta on mitochondria (indicating the formation of phagophore-like structures) before uptake into early endosomes. Hence, mitochondria are likely delivered to early endosomes in a distinct way in the ATG16L1[100-250]-driven mitophagy pathway.

Though we have induced this unconventional mitophagy pathway in a somewhat artificial manner, given its robust response we believe it will have physiological significance. Indeed, Atg8-family protein conjugation-independent autophagy and mitophagy has previously been identified and bears hallmarks similar to the pathway discovered here. The small GTPase RAB9, which is involved in endolysosomal trafficking, has been associated with an Atg8-family protein conjugation-independent mitophagy pathway that still requires the ULK1 and the PtdIns3K complexes. Here, mitochondria are alternatively enclosed in RAB9-positive membranes [79–84]. Importantly, this RAB9-dependent alternative mitophagy pathway was shown to occur under physiological conditions, such as during different stress conditions in the heart where mitophagy plays a protective role [82–84] or during iPSC reprogramming [81]. Therefore, RAB9 is an ideal candidate to test in the ATG16L1[100-250]-driven mitophagy pathway identified here and will be a focus of future work.

### Outlook

In this study, we identified possible candidates for targeted organelle degradation. We found that the recruitment of several core autophagy proteins (ULK1, ATG16L1, different LC3 proteins) to mitochondria using a nanobody is capable and sufficient to induce mitophagy. Furthermore, depending on which autophagy proteins were recruited, mechanistically different pathways were induced. Thus, targeted organelle degradation by directly recruiting different core autophagy proteins to mitochondria could be a novel strategy for treating diseases where the removal of dysfunctional mitochondria could be advantageous, such as in PD. Excitingly, the development of small molecule “mitochondrial recruiters” is already underway. Heterobifunctional molecules have been developed that bind to the cargo (e.g. mitochondria) on one side and on the other side target a component of the autophagy machinery. Thereby, current degraders are primarily based on interacting with either autophagy receptors or Atg8-family proteins to induce delivery of their cargo to lysosomes. The Autophagy-Targeting Chimera (AUTAC) is a heterobifunctional degrader based on a guanine derivative that primes the cargo for K63-linked ubiquitination, recognition by autophagy receptors (e.g., SQSTM1) and subsequent lysosomal delivery. The AUTAC system was shown to be efficient to induce the lysosomal degradation of soluble proteins. However, a mitochondrially targeted AUTAC was not sufficient on its own to induce mitophagy but could induce mitophagy in cells with fragmented and damaged mitochondria, such as upon knockdown of OPA1 (a regulator of mitochondrial inner membrane fusion) or upon CCCP treatment [2]. The Autophagy-Targeting Chimera (AUTOTAC) is a similar heterobifunctional degrader molecule that uses an SQSTM1-binding module to recruit the autophagy machinery. Lysosomal degradation of soluble proteins and of protein aggregates was demonstrated using the AUTOTAC system [8], whereas degradation of organelles (including mitochondria) was not reported so far. The third major heterobifunctional degrader system links cargo to Atg8-family proteins and thus tethers cargo to phagophores. Hence, this degrader was termed Autophagosome-Tethering Compound (ATTEC). To date, lysosomal degradation of protein aggregates, lipid droplets and soluble proteins (but not mitochondria) was demonstrated using ATTECs [3–6]. Beyond these heterobifunctional chemical degraders, a recent study found that tagging pathogenic HTT (huntingtin) with the ATG16L1-binding region of TMEM59 (transmembrane protein 59) or the LIR motif of SQSTM1 was sufficient to induce lysosomal degradation of Huntingtin aggregates. Moreover, targeting the ATG16L1-binding region or the LIR motif to mitochondria, by fusing them to the mitochondrial targeting sequence of the outer mitochondrial membrane protein TOMM20, induced moderate degradation of mitochondria in the presence of FCCP or upon concomitant EBSS treatment [7]. Our results here suggest that multiple different autophagy proteins can be recruited to mitochondria to induce mitophagy, which hopefully increases the potential pool of chemical matter that could be used for therapeutically targeting mitophagy.

## Materials and methods

### Antibodies

Primary antibodies for immunoblotting (IB) or immunofluorescence staining (IF) were used as following: anti-ATG5 (Cell Signalling Technology 12994; rabbit; 1:1000 for IB), anti-ATG13 (Merck, SAB4200100; rabbit; 1:2000 for IB), anti-COX4/COX IV (Cell Signalling Technology, 4850S; rabbit; 1:1000 for IB, 1:500 for IF), anti-ATG16L1 (Abcam 187671; rabbit; 1:1000 for IB), anti-RB1CC1/FIP200 (Proteintech 17250–1-AP; rabbit; 1:1000 for IB), anti-FLAG (Merck, F1804; mouse; 1:1000 for IB, 1:200 for IF), anti-GAPDH (Proteintech 60004–1-Ig; mouse; 1:10000 for IB), anti-GFP (Roche 11814460,001; mouse; 1:1000 for IB), anti-HA (Merck 11867423001; rat; 1:500 for IF), anti-HSPD1/HSP60 (Cell Signalling Technology, 4870; rabbit; 1:1000 for IB), anti-LAMP1 (Santa Cruz Biotechnology, sc -20011; mouse; 1:200 for IF), anti-LC3A/B (Cell Signalling Technology, 4108; rabbit; 1:1000 for IB), anti-HTRA2/OMI (MRC PPU Reagents and Services, University of Dundee; sheep; 1:1000 for IB), anti-OPA1 (BD Biosciences 612606; mouse; 1:2000 for IB), anti-TUBA/α-tubulin (Proteintech, 6603–1-1 g; mouse; 1:10000 for IB), anti-ULK1 (Cell Signalling Technology, 8054; rabbit; 1:1000 for IB; 1:250 for IF), anti-VCL/vinculin (Abcam, ab129002; rabbit; 1:5000 for IB), and anti-WIPI2 (Bio-Rad, MCA5780GA; mouse, 1:500 for IF).

Secondary antibodies for IB were used as following: goat anti-mouse IgG (H+L) HRP (Thermo Scientific 31430; 1:5000), goat anti-rabbit IgG (H+L) HRP (Thermo Scientific 31460; 1:5000), and donkey anti-sheep IgG (H+L) HRP (Thermo Scientific, A16041; 1:5000).

Secondary antibodies for IF were used as following: goat anti-mouse Pacific Blue (Thermo Scientific, P31582; 1:500), goat anti-mouse Alexa Fluor 488 (Thermo Scientific, A-21121; 1:1000), goat anti-rabbit Pacific Blue (Thermo Scientific, P-10994; 1:500), goat anti-rat Alexa Fluor 488 (Thermo Scientific, A-11006; 1:1000), and goat anti-rabbit Alexa Fluor 488 (Thermo Scientific, A-11008; 1:1000).

## Plasmids

VSV-G and GAG-POL (Clontech/TakaraBio, 6161, 63152) and pBABE mCherry-GFP-SKL were used as previously described [69]. The following plasmids were obtained from MRC PPU Reagents and Services, University of Dundee: pBABE mCherry-GFP-FIS1[101-152] (*mito*-QC reporter) (DU40799), pBABE COX8[1–36]-COX8[1–36]-mCherry-GFP (*matrix*-QC reporter) (DU65070), pBABE mCherry-FIS1[101-152] (DU55314), pBABE mCherry-GFP-PEX26[245-274] (DU59357), pBABE FLAG anti-GFP[6mut] (DU57701), pBABE FLAG anti-mCherry (DU54282), pBABE FLAG anti-GFP[6mut]-ULK1 (DU59808), pBABE FLAG anti-mCherry-ULK1 (DU65158), pBABE FLAG ULK1 (DU45617), pBABE FLAG ATG16L1 (DU65742), pBABE FLAG anti-mCherry-ATG16L1 (DU71361), pBABE FLAG anti-mCherry-ATG16L1[100-250] (DU65949), pBABE FLAG anti-mCherry-ATG16L1[1-79] (DU71362), pQCXIP FLAG anti-mCherry-GABARAP (DU71897), pQCXIP FLAG anti-mCherry-GABARAPL1 (DU71898), pQCXIP FLAG anti-mCherry-GABARAPL2 (DU71899), pQCXIP FLAG anti-mCherry-LC3A (DU71906), pQCXIP FLAG anti-mCherry-LC3B (DU71907), pQCXIP FLAG anti-mCherry-LC3C (DU71908), pQCXIP FLAG anti-mCherry (DU71896), pBABE RB1CC1/FIP200 (DU55995), pBABE GFP-WIPI2b (DU72701), pBABE 3×HA-ATG16L1 (DU78042) and pBABE HA-RAB5A^Q79L^ (DU71545).

pBABE FLAG anti-mCherry-ULK1^K46I^ was modified from pBABE FLAG anti-mCherry-ULK1 (DU65158), pBABE FLAG anti-GFP[6mut]-ULK1^K46I^ was modified from pBABE FLAG anti-GFP[6mut]-ULK1 (DU59808) and pBABE FLAG anti-mCherry-ATG16L1[100–201], pBABE FLAG anti-mCherry-ATG16L1[207–230], pBABE FLAG anti-mCherry-ATG16L1[229–246], pBABE FLAG anti-mCherry-ATG16L1[100–250]^E226R^, pBABE FLAG anti-mCherry-ATG16L1[100–250]^E241R^, pBABE FLAG anti-mCherry-ATG16L1[100–250]^E226R,E241R^, pBABE FLAG anti-mCherry-ATG16L1[202–250], pBABE FLAG anti-mCherry-ATG16L1[202–250]^E226R^, pBABE FLAG anti-mCherry-ATG16L1[202–250]^E241R^ and pBABE FLAG anti-mCherry-ATG16L1[202–250]^E226R,E241R^ were modified from pBABE FLAG anti-mCherry-ATG16L1[100–250] (DU65949) using the In-Fusion HD cloning kit (TakaraBio 102518).

## Cell culture

ARPE-19 cells (ATCC, CRL‐2302) were cultured in a 1:1 mix of DMEM and Ham’s F-12 Nutrient Mix (Thermo Scientific 21331020) supplemented with 10% (v:v) FBS, 2 mM L-glutamine (Thermo Scientific 25030024), 100 U/ml penicillin and 0.1 mg/ml streptomycin (Thermo Scientific 15140122). HEK293FT cells, immortalized mouse embryonic fibroblasts (MEFs; *atg5* KO MEFs were a kind gift from Prof. Noboru Mizushima, Tokyo Medical and Dental University [85]; *atg16l1* CRISPR KO MEFs were a kind gift from Dr Noor Gammoh, University of Edinburgh), A549 cells (WT and *ATG5* CRISPR KO cells were a kind gift from Prof. Simon Wilkinson, University of Edinburgh) were cultured in DMEM (Thermo Scientific 11960044) supplemented with 10% (v:v) FBS, 2 mM L-glutamine, 100 U/ml penicillin and 0.1 mg/ml streptomycin. All cells were cultured in humidified incubators at 37°C and 5% CO_2_. Cell lines were regularly tested for mycoplasma (MycoAlert Detection kit; Lonza; LT07–318).

### Generation of CRISPR KO cells

*RB1CC1* KO ARPE-19 cells were generated using CRISPR-Cas9 technology. WT ARPE-19 cells were co-transfected with Cas9 D10A nickase (Cas9n) and sense (GATACCGCAGATGCTGAAAG) and antisense (GACACATGTTCCACTGACTT) guide RNAs targeting exon 7 of *RB1CC1* (obtained from MRC PPU Reagents and Services; University of Dundee: DU69707 [pBabe P U6 with sense guide] and DU69709 [pX335 with anti-sense guide and Cas9n D10A]). For this, 250 µl Opti-MEM (Thermo Scientific 31985062) were mixed with 48 µl GeneJuice Transfection Reagent (Merck 70967–3) and incubated for 5 min at room temperature (RT) before adding 4  μg of pBabe P U6 encoding the sense guide and 4 μg of pX335 encoding the anti-sense guide and Cas9n D10A and incubating for 20 min at RT. The mix was then added dropwise to ARPE-19 WT cells in a 6-cm dish at a confluency around 60%. After 24 h, cells were selected with puromycin (2 μg/ml) for 48 h and then allowed to recover. Single-cell FACS sorting was performed and individual single cell clones were analyzed by immunoblotting and DNA sequencing.

### Retrovirus generation and viral transduction

For the generation of retroviral particles, HEK293FT cells were co-transfected with 6 µg of the cDNA of interest (pBABE or pQCXIP vector), 3.8 µg GAG-POL and 2.2 µg VSV-G per 10 cm dish by first mixing the three plasmids with 36 µl of polyethylenimine/PEI (1 mg/ml in 50 mM HEPES, pH 7.4) and 1 ml of serum-free DMEM containing 100 U/ml penicillin and 0.1 mg/ml streptomycin for 20–30 min and then adding the mix dropwise on HEK293FT cells in a 10-cm dish at a confluence of 60% and cultured in 5 ml serum-free DMEM containing 100 U/ml penicillin and 0.1 mg/ml streptomycin. After 5 h of incubation at 37°C, the medium was replaced with fresh 10 ml DMEM containing 10% FBS, 2 mM L-glutamine, 100 U/ml penicillin and 0.1 mg/ml streptomycin. The medium was changed again 24 h after the transfection. Retroviral particle-containing supernatant was harvested 48 h and 72 h after the transfection and passed through a 0.45-µm filter. The cell line of interest was transduced with the retroviral particle-containing supernatant in the presence of polybrene (hexadimethrine bromide; 10 µg/ml). For transient transduction of cells with retroviral particles, cells were transduced for the indicated time without selection before harvesting. In order to generate stable cell lines, cells were transduced with retroviral particle-containing supernatant for 24 h before refreshing the medium and selection of cells expressing the protein of interest with 2 μg/ml puromycin (Merck, P4512) or 800 μg/ml hygromycin (Thermo Scientific 10687010). Cells were selected with the respective antibiotic until all non-transduced cells in a control dish were dead.

### Treatment of cells with drugs

Cells were treated as indicated with deferiprone (1 mM; 3‐hydroxy‐1,2‐dimethyl‐4(1 H)‐pyridone) (Merck 379409), VPS34-IN1 (1 µM; MRC PPU Reagents and Services, University of Dundee), bafilomycin A_1_ (50 nM; Enzo, BML‐CM110), or CCCP (10 μM; Merck, C2759).

### Fluorescence reporter assay (microscopy)

Cells stably expressing the *mito*-QC reporter (mCherry-GFP-FIS1[101-152]), the *matrix*-QC reporter (COX8[1–36]-COX8[1–36]-mCherry-GFP) or *pexo*-QC reporters (mCherry-GFP-PEX26[245-274] or mCherry-GFP-SKL) were grown on coverslips in 12 wells or 24 wells. For harvesting, cells were washed twice with Dulbecco’s PBS (DPBS; Thermo Scientific, H1399) and then fixed in 3.7% (w:v) paraformaldehyde (PFA) in 200 mM HEPES pH 7.0 for 10 min. Samples were washed twice and then incubated for 10 min with IF quenching buffer (DMEM with 10 mM HEPES, pH 7.4, 0.02% [w:v] NaN_3_). Samples were washed once with DPBS. Nuclei were stained with Hoechst 33342 (1 μg/ml in DPBS) for 5 min. Samples were washed three times with DPBS before mounting (ProLong Glass Antifade Mountant; Invitrogen, P36984). Images were taken using a Nikon Eclipse Ti widefield microscope (60× objective) unless otherwise stated. Images were analyzed using FIJI. The quantification of the mean number of mCherry^+^ GFP^−^ mitolysosomes (as defined as puncta with high mCherry fluorescence but no corresponding GFP fluorescence) per cell (based on the *mito*-QC or *matrix*-QC reporters) was performed in a semi-automated way using the FIJI macro “*mito*-QC counter” as described previously [86]. The number of mCherry^+^ GFP^−^ pexolysosomes (as defined as puncta with high mCherry fluorescence but no corresponding GFP fluorescence) per cell (based on the mCherry-GFP-PEX26[245-274] or mCherry-GFP-SKL reporters) was quantified manually. The mean number of mitolysosomes or pexolysosomes per cell was calculated from all analyzed cells for each individual independent experimental replicate and the mean ± standard deviation of the means of the individual independent experimental replicates was displayed in bar graphs. All quantifications were performed using unprocessed images. For representative purposes, the intensity of the images was adjusted.

### Immunofluorescence (IF) staining

Cells were grown on coverslips in 12 wells or 24 wells. For harvesting, cells were washed twice with DPBS and then fixed in 3.7% (w:v) PFA in 200 mM HEPES, pH 7.0 for 10 min. Samples were washed twice and then incubated for 10 min with IF quenching buffer (DMEM with 10 mM HEPES, pH 7.4, 0.02% [w:v] NaN_3_). Samples were washed once with DPBS and then permeabilized by incubating with permeabilization buffer (0.2% [v:v] NP-40 [Merck 492016] in PBS) for 3 min. Cells were washed twice and then incubated for 30 min with IF blocking buffer (1% [w:v] BSA [Merck 10735108001] in PBS, 0.02% [w:v] NaN_3_) on a shaker. Samples were probed with primary antibodies (diluted in IF blocking buffer) for 1 h at 30°C in a humidified chamber. Samples were washed 3 times for 10 min with IF blocking buffer before staining with a fluorophore-coupled secondary antibody (diluted in IF blocking buffer) for 30 min at RT. Samples were washed 3 times for 10 min with IF blocking buffer. If nuclei were stained, cells were incubated with Hoechst 33342 (1 μg/ml in DPBS) for 5 min and washed three times in DPBS. Cells were mounted using ProLong Glass Antifade Mountant (Invitrogen, P36984). Images were taken using the Zeiss LSM880 Airyscan Confocal Scanning microscope (63× or 100× objectives). Images were analyzed using FIJI. Quantification of puncta or quantification of RAB5A^Q79L^-positive enlarged early endosomes containing mitochondria was performed manually. Displayed is the mean ± standard deviation of the mean number of puncta of the individual independent experimental replicates. The intensity of images was adjusted for representative purposes.

### MitoTracker Deep Red staining

Cells were grown on coverslips in 24 wells. Before harvesting, cells were incubated with MitoTracker Deep Red FM (Invitrogen, M22426; 100 nM in normal cell culture medium) for 30 min at 37°C. Cells were then washed twice with DPBS and fixed in 3.7% (w:v) PFA in 200 mM HEPES, pH 7.0 for 10 min. Subsequent immunofluorescence staining was performed as described above.

Quantification of MitoTracker Deep Red staining was performed as following: the intensity of Mitotracker Deep Red above a background threshold was quantified from line plots using FIJI and normalized to the mCherry intensity above a background threshold from the same line plot.

### Flow cytometry analysis of *mito*-QC

Cells stably expressing the *mito*-QC reporter or, as a control, WT cells that do not express the *mito*-QC reporter were grown in 6-cm dishes. To harvest the cells, cells were trypsinized, centrifuged (300 × g, 3 min) and the cell pellet was resuspended in 50 µl DPBS. Cells were fixed by adding 1.0 ml 3.7% (w:v) PFA in 200 mM HEPES, pH 7.0 for 30 min at RT. After adding 2.5 ml DPBS, cells were centrifuged (300 × g, 3 min) and the cell pellet was resuspended in DPBS containing 1% FBS and transferred to FACS tubes. Samples were analyzed using a LSR Fortessa (BD). For each experimental replicate 50,000 cells in the cell population visible in the FSC-A and SSC-A profiles were acquired. Samples were analyzed using FlowJo10 as following: (1) Cells were gated for based on the FSC-A and SSC-A profiles. (2) Single cells were gated for based on the FSC-W and FSC-A profiles. (3) Cells expressing the *mito*-QC reporter were gated for based on the GFP and mCherry channels using WT cells that do not express the *mito*-QC reporter as a negative control and applying this gate to all other samples. (4) The percentage of mitophagic cells was determined by applying the gate for mitophagic cells of not treated control samples to all other samples. The mean ± standard deviation of the percentage of mitophagic cells of the individual independent experimental replicates is displayed in bar graphs.

### Cell lysis

For cell lysis, cells were washed twice with cold PBS on ice. NP-40 lysis buffer (50 mM HEPES pH 7.4, 150 mM NaCl, 1 mM EDTA, 10% glycerol, 0.5% NP-40, 1 mM DTT [added prior to use], 1× phosphatase inhibitor cocktail (115 mM sodium molybdate [Merck 243655], 400 mM sodium tartrate dihydrate [Merck 1066640100],1 M β-glycerophosphate disodium salt pentahydrate [Merck 35675], 100 mM sodium fluoride [Merck 201154], 100 mM sodium orthovanadate [Merck, S6508; added prior to use], 1× EDTA-free protease inhibitor cocktail [Roche 11873580001; added prior to use]) was added to the cells, cells were scraped and incubated on ice for 20 min. The cell lysates were cleared at 20000 g for 10 min at 4°C. The protein concentration of the cell lysates was determined using the Bradford assay (Bio-Rad 5000006) according to the manufacturer’s instructions. Protein concentrations were adjusted between samples.

### Immunoprecipitation (IP)

#### IP of the *mito*-QC reporter (anti-mCherry)

For immunoprecipitation of the *mito*-QC reporter, mCherry binder sepharose beads (obtained from MRC PPU Reagents and Services, University of Dundee) were washed 3 times with cold PBS (MRC PPU Reagents and Services, University of Dundee) directly before use (200 g, 1 min). Equal amounts of cell lysates (obtained as described above) were incubated with the mCherry binder sepharose beads overnight at 4°C on a rotating wheel (20 µl slurry mCherry binder sepharose beads for every 1 mg of total protein). Samples were washed 3 times with NP-40 lysis buffer (1 min, 200 g, 4°C). For elution, the beads were incubated with 30 µl of 2× LDS-sample buffer containing 2.5% β-mercaptoethanol for 5 min at 95°C. The beads were then centrifuged down at 20000 g for 1 min and the eluate-containing supernatant was collected and cleared from remaining beads (Corning Costar Spin-X centrifuge tube filter 0.22 µm [Merck, CLS8161]; 20000 g, 1 min).

#### IP of FLAG-tagged proteins:

For IP of FLAG-tagged anti-mCherry-ULK1, cells were lysed using NP-40 lysis buffer as described above. Anti-FLAG M2 affinity gel (MRC PPU Reagents and Services, University of Dundee) was washed 3 times with cold PBS and once with NP-40 lysis buffer directly before use. Equal amounts of cell lysates were incubated with the anti-FLAG affinity gel (20 µl slurry anti-FLAG agarose gel for every 1 mg of total protein) overnight at 4°C on a rotating wheel. Samples were washed 3 times (1 min at 200 g at 4°C). For the first wash, NP-40 lysis buffer containing 450 mM NaCl was used. For the second and third wash, NP-40 lysis buffer containing 150 mM NaCl was used. Bound protein was eluted from the anti-FLAG affinity gel by incubating with 30 µl of 1× LDS-sample buffer (without β-mercaptoethanol) for 5 min at 95°C. The anti-FLAG affinity gel was then centrifuged down at 20,000 g for 1 min, the eluate-containing supernatant was cleared from remaining beads (Corning Costar Spin-X centrifuge tube filter 0.22 µm; 20000 g, 1 min) and 1% β-mercaptoethanol was added.

### Immunoblotting

Equal amounts of protein (5–15 µg) from cell lysates containing 1× SDS sample buffer (62.5 mM Tris, pH 6.8, 10% glycerol, 2% SDS, 0.005% bromophenol blue, 10% [v:v] β-mercaptoethanol) were boiled for 5 min at 95°C and then resolved on gradient 6–15% Bis-Tris gels by SDS-PAGE. Proteins were subsequently transferred onto Amersham Protran nitrocellulose membranes (0.45 µm; Cytiva 10600041). Total protein on the membrane was stained with Ponceau S, before blocking for 1 h at RT in 5% milk in TBS (MRC PPU Reagents and Services, University of Dundee) supplemented with 10% Tween-20 (Merck, P1379; TBST). Membranes were probed with primary antibody (diluted in 5% BSA in TBST [exception: anti-HTRA2/OMI antibody which was diluted in 5% milk in TBST]) overnight at 4°C. After washing three times with TBST, membranes were probed with HRP-conjugated secondary antibodies (diluted in 5% milk in TBST) for 1 h at RT before washing three times with TBST. The respective signals were detected using Clarity Western ECL Substrate Solution (Bio-Rad 1705062) and the ChemiDoc Imaging System (Bio-Rad). For quantification, band densitometry analysis was performed using FIJI. Protein levels were normalized to a loading control from the same membrane and the mean ± standard deviation of the individual independent experimental replicates is displayed in bar graphs.

### CS activity assay

CS activity was measured in cell lysates according to the method of [87]. Cell lysates (2 µl) were incubated with 196 µl CS buffer (100 mM Tris, pH 8.0; 0.1% Triton X-100 [Merck, T8787]; 0.1 mM acetyl-CoA [Merck, A2056]); 0.2 mM DTNB [5“, 5”-Dithiobis 2-nitrobenzoic acid; Merck, D8130]) in quadruplicates in a 96-well plate. To start the assay, 2 µl oxaloacetate (final concentration 0.2 mM; Merck, O4126) were added. Samples without addition of oxaloacetate were used as a reference. Absorbance was measured at 412 nm every 30 s for 60 min at 30°C using a PHERAstar FSX microplate reader (BMG Labtech). Citrate synthase activity was calculated as following:

$$\mathrm{Citrate}\;\mathrm{synthase}\;\mathrm{activity}\left[\frac{\mu \mathrm{mol}}{\min\times \mathrm{mg}}\right]=\frac{\frac{\Delta \;\mathrm{absorbance}\left(412\mathrm{nm}\right)}{\mathrm{time}\left[\min\right]}x\;\mathrm{volume}\;\mathrm{in}\;\mathrm{well}\left[\mathrm{ml}\right]}{\mathrm{extinction}\;\mathrm{coefficient}\left[M^{-1}\times \;cm^{-1}\right]\times \;\mathrm{pathlength}\left[\mathrm{cm}\right]x\;\mathrm{protein}\;\mathrm{amount}\left[\mathrm{mg}\right]}$$

with: extinction coefficient of DTNB: 13.6 M^−1^ cm^−1^.

The mean ± standard deviation of the individual independent experimental replicates is displayed in bar graphs.

### Transmission electron microscopy (TEM)

Cells grown in 10-cm dishes were fixed by adding 0.1 M sodium cacodylate buffer (pH 7.2) containing 4% PFA and 2.5% glutaraldehyde (Agar Scientific, AGR1020) on top of cells for 30 min at RT before cells were scraped into the fixing buffer and incubated for another 30 min. Cells were washed twice with 0.1 M sodium cacodylate (Agar Scientific, AGR1503) buffer and then incubated with sodium cacodylate buffer containing 1% osmium tetroxide (Agar Scientific, AGR1016) and 1.5% sodium ferrocyanide (Merck 13425) for 60 min. Samples were incubated with 1% tannic acid in 0.1 M sodium cacodylate buffer for 1 h and washed with sodium acetate buffer (pH 5.0; 0.2 M) overnight before staining with 1% uranyl acetate (Agar Scientific, AGR1000) in sodium acetate buffer for 1 h. Samples were dehydrated with a sequential series of increasing alcohol concentrations (from 50% to 100%) with 10 min incubations each. Ethanol was then removed by washing twice for 10 min with 100% propylene oxide (Agar Scientific, AGR1080). The samples were then incubated with 50% propylene oxide:50% Durcupan resin (Merck 44611) overnight in a rotator before propylene oxide was allowed to evaporate. Samples were put in fresh 100% Durcupan resin and allowed to polymerize at 60°C overnight. Sections of a thickness of 70–100 nm were cut using an ultramicrotome (Leica Ultracut UCT). Sections were stained with 3% aqueous uranyl acetate followed by Reynolds lead citrate staining for 10 min each. Grids were imaged on a JEOL 1200EX TEM using a SIS camera.

### Statistical analysis

Statistical analysis was performed in Graph Pad Prism 9 from at least three biologically independent experimental replicates. Displayed is the mean ± standard deviation. For comparison of two different conditions an unpaired two-tailed t-test was used. For comparisons of multiple conditions, a One-Way (1 variable) or Two-Way (2 different variables) ANOVA with Dunnett’s or Tukey’s multiple comparisons post-hoc test were used as indicated. Statistical significance is denoted as: ns = not significant, *= *p* < 0.05, **= *p* < 0.01, ***= *p* < 0.001, ****= *p* < 0.0001.

## Supplementary Material

Supplemental Material
