# Supplementary material for: Artificial targeting of autophagy components to mitochondria reveals both conventional and unconventional mitophagy pathways
Source: Autophagy. 2024 Aug 23;21(2):315–37. doi: 10.1080/15548627.2024.2395149 (PMC11760219; doi:10.1080/15548627.2024.2395149)
Supplement: Supplemental Material [file KAUP_A_2395149_SM6368.docx]

**Artificial targeting of autophagy components to mitochondria reveals both conventional and unconventional mitophagy pathways**

Katharina C. Lorentzen^1^, Alan R. Prescott^2^ and Ian G. Ganley^1*^

^1^ MRC Protein Phosphorylation and Ubiquitylation Unit, University of Dundee, Dundee DD1 5EH, UK

^2^ Dundee Imaging Facility, School of Life Sciences, University of Dundee, Dundee DD1 5EH, UK

* Address for correspondence: [i.ganley@dundee.ac.uk](mailto:i.ganley@dundee.ac.uk)

# **Supplemental Figures**


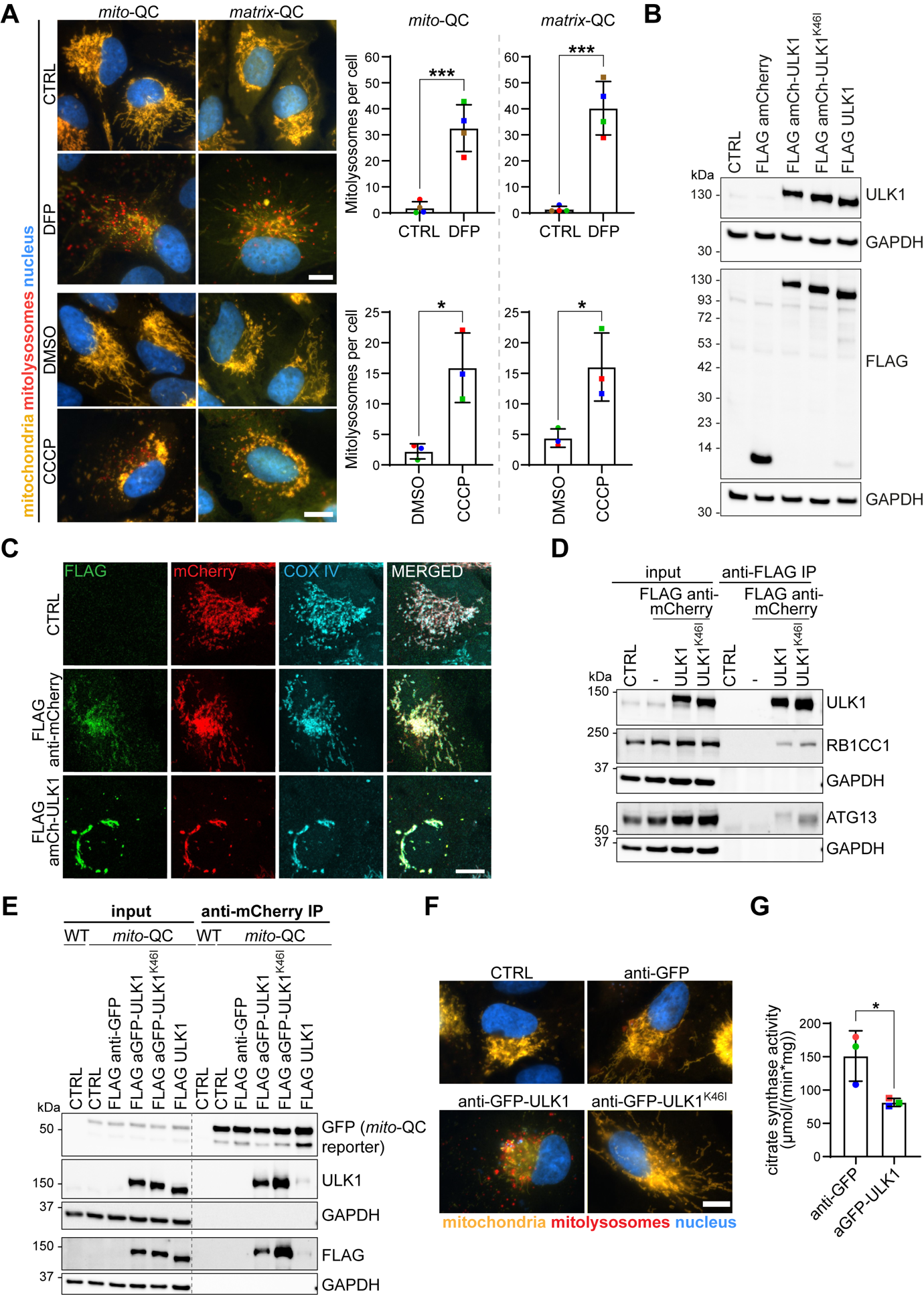


**Figure S1.** Targeting exogenous ULK1 to mitochondria induces mitophagy. (**A**) ARPE-19 *mito*-QC or *matrix*-QC cells were not treated (CTRL) or treated with DFP (1 mM; upper panel), or were treated with DMSO (0.1%) or CCCP (10 μM; lower panel), for 24 h before widefield microscopy analysis. Left: Representative images. Scale bar: 10 µm. Right: Quantification of mitophagy showing the mean number of mitolysosomes per cell from 3-4 independent experiments with a minimum of 47 cells being analyzed for each condition in each experiment. Statistics: Unpaired Two-tailed t-test. (**B**) ARPE-19 *mito*-QC cells were transduced to express the indicated proteins or were not transduced (CTRL) for 4 days before cell lysis and immunoblot analysis. (**C**) ARPE-19 cells stably expressing mitochondrially localized mCherry-FIS1[101-152] were transduced (or not – CTRL) to express the indicated FLAG-tagged proteins for 48 h before immunofluorescence staining using anti-FLAG and anti-COX4 (mitochondrial protein) antibodies and analysis by confocal microscopy. Scale bar: 10 µm. (**D**) ARPE-19 *mito*-QC cells were transduced with the indicated proteins, or not (CTRL), for 24 h before cell lysis and immunoprecipitation using anti-FLAG beads. Co-IP of the other components of the ULK1 complex was analyzed by immunoblotting. Input: 5.7% of IP. (**E**) ARPE-19 WT cells or ARPE-19 *mito*-QC cells were transduced with the indicated proteins for 24 h, or not transduced (CTRL), before cell lysis. The *mito*-QC reporter was immunoprecipitated using anti-mCherry beads followed by immunoblotting. Input: 4.3% of IP. (**F**) ARPE-19 *mito*-QC cells were transduced with the indicated proteins for 48 h before widefield microscopy analysis. Shown are representative images. Scale bar: 10 µm. (**G**) ARPE-19 *mito*-QC cells were transduced with the indicated proteins for 72 h. Cells were lysed and citrate synthase activity in lysates was measured. Shown is the quantification of 3 independent experiments. Statistics: Unpaired two-tailed t test.


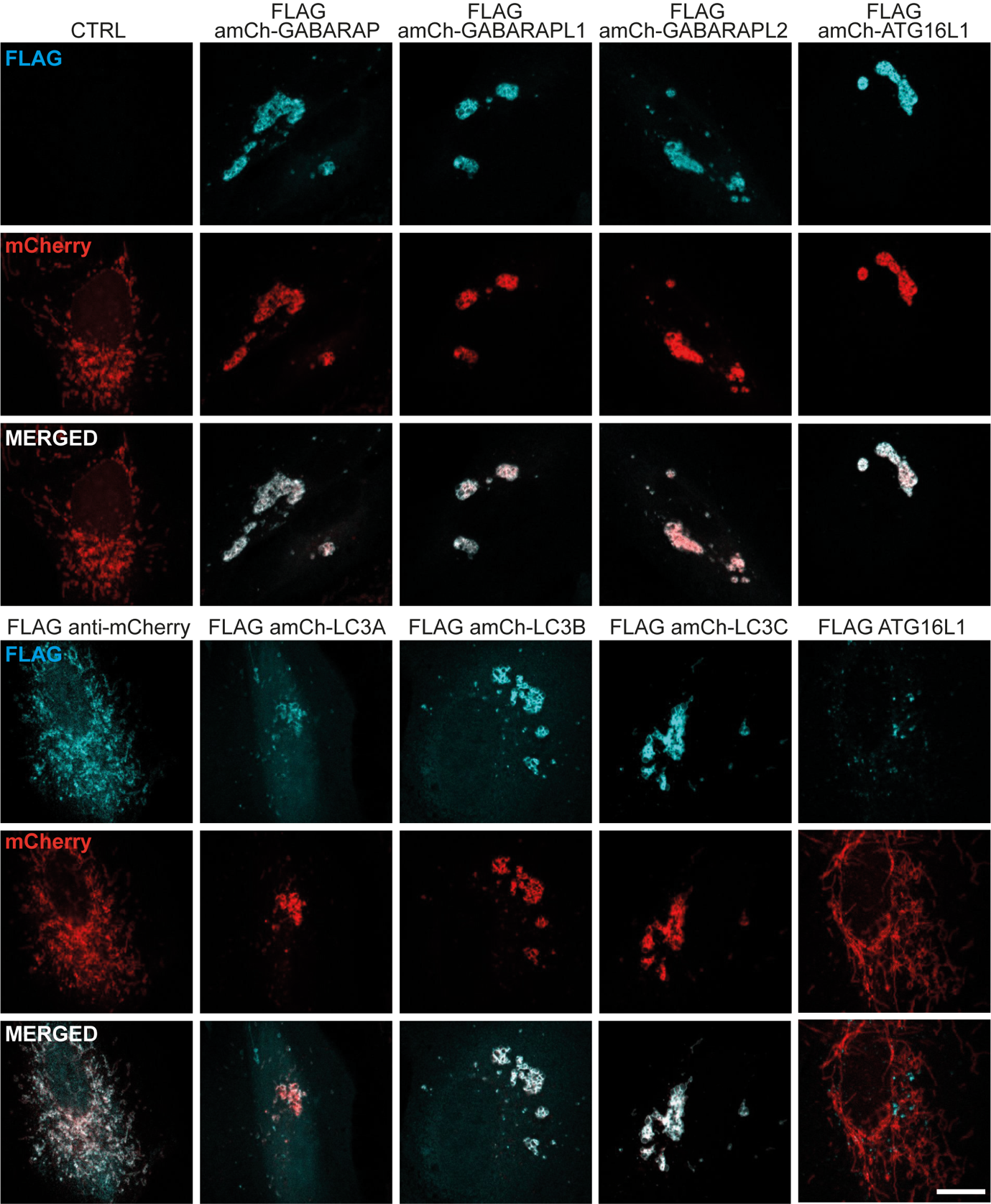


**Figure S2.** Localization of anti-mCherry-ATG8s and anti-mCherry-ATG16L1. ARPE-19 cells stably expressing mitochondrial localized mCherry-FIS1[101-152] were transduced with the indicated proteins or were not transduced (CTRL) for 48 h before immunofluorescence staining using an anti-FLAG antibody and analysis by confocal microscopy. Shown are representative images. Scale bar: 10 µm.


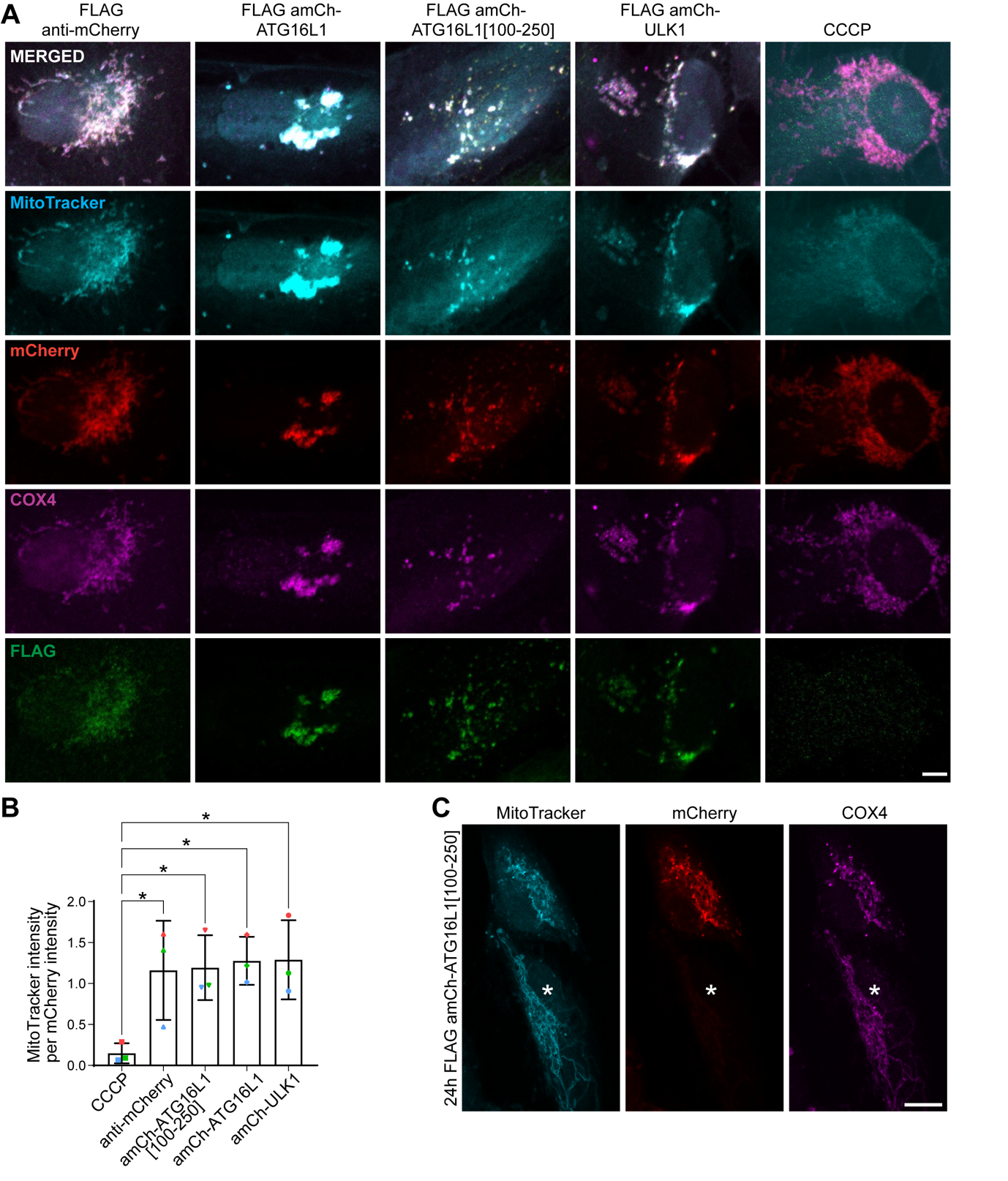


**Figure S3.** Mitochondrial membrane potential is maintained upon nanobody-mediated mitochondrial recruitment of different autophagy proteins. (**A**) ARPE-19 cells stably expressing mitochondrial localized mCherry-FIS1[101-152] were transduced to express the indicated FLAG-tagged proteins for 48 h. Alternatively, cells were treated with CCCP (10 μM) for 24h. Cells were stained with MitoTracker Deep Red for 30 min before fixing. Subsequently, immunofluorescence staining using an anti-FLAG and anti-COX4 antibody was performed. Scale bar: 5 µm. (**B**) Quantification of the intensity of MitoTracker Deep Red normalized to the intensity of mitochondrial mCherry from 3 independent experiments. At least 5 cells were analyzed per condition per experimental replicate. Statistics: One-Way ANOVA + Dunnett’s multiple comparisons test (comparison to CCCP). (**C**) ARPE19 cells expressing mCherry-FIS1[101-152], or not (marked with white asterisk) were transduced to express anti-mCherry-ATG16L1[100-250] for 24 h (to confirm no mitochondrial depolarization at an earlier time point than in A and B). Cells were stained as in panel A. Scale bar: 10 µm. Note that cell without mCherry-FIS1[101-152] cannot recruit the ATG16L1 construct and has similar MitoTracker staining to the one that can.


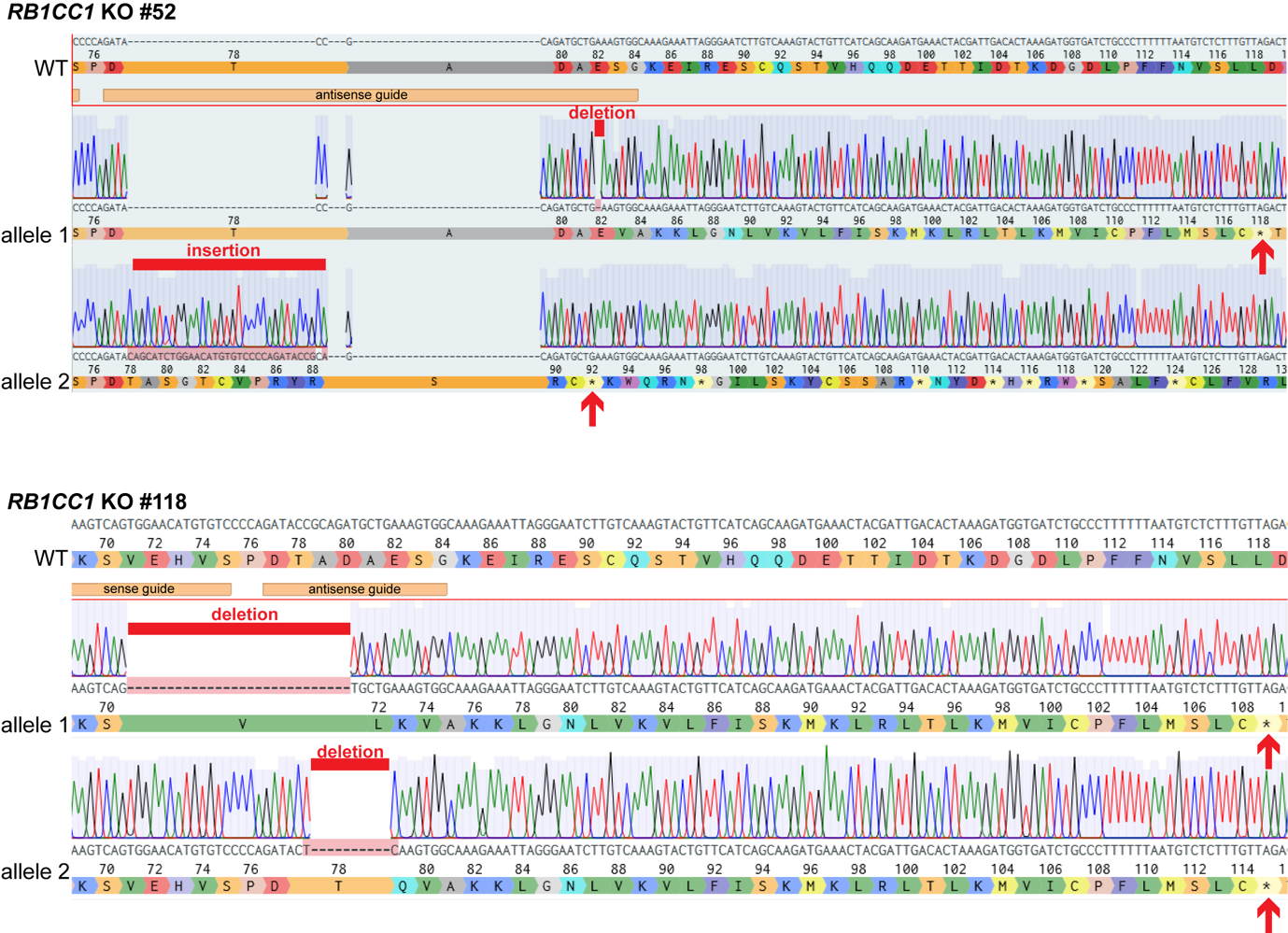


**Figure S4.** Sequence analysis of two distinct *RB1CC1* KO clones. Two distinct *RB1CC1* KO clones (#52 and #118) were generated in diploid ARPE19 cells using CRISPR-Cas9 technology targeting exon 7 of *RB1CC1*. Shown are the sequence alignment data for both clones. In *RB1CC1* KO #52, a deletion of a single base in allele 1 and an insertion in allele 2 result in a frameshift and premature stop codons (marked with red arrows) in both alleles. In *RB1CC1* KO #118, two distinct deletions in allele 1 and allele 2 result in a frameshift and premature stop codons (marked with red arrows) in both alleles.


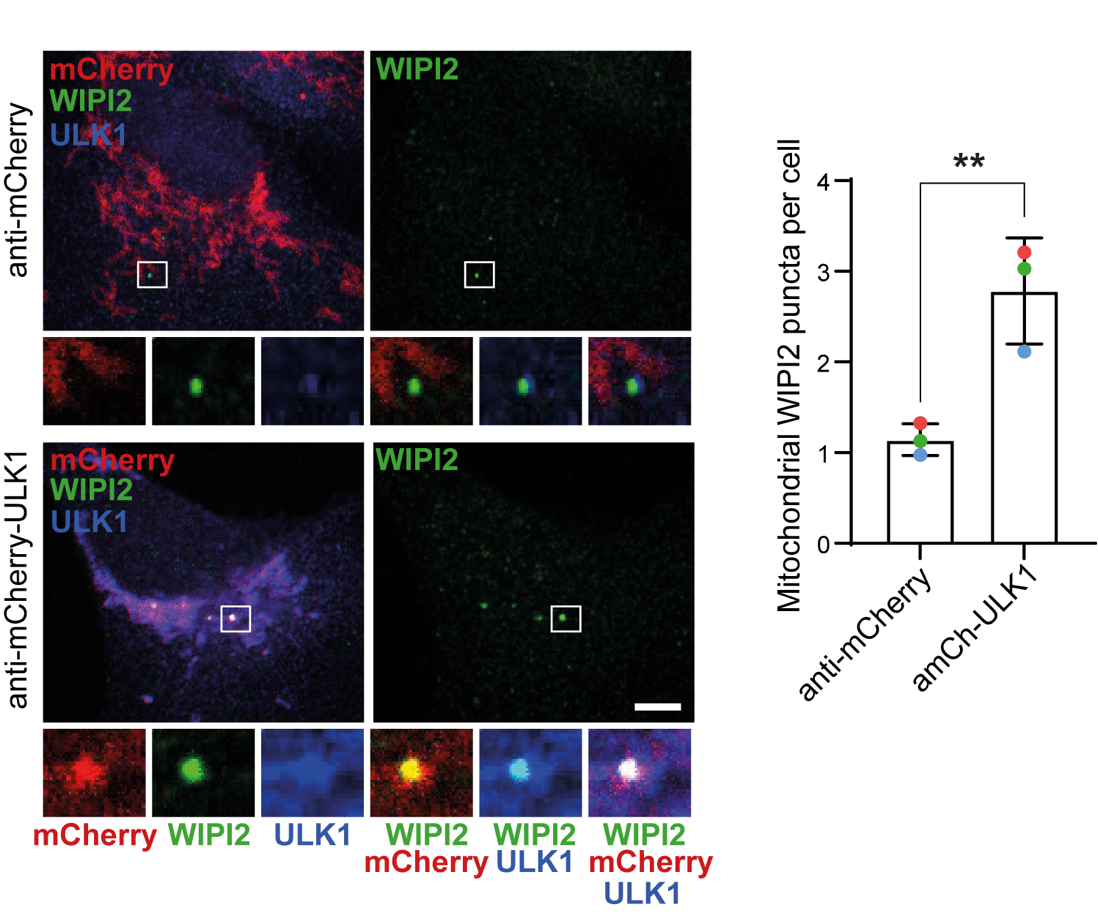


**Figure S5.** Mitochondrial WIPI2 puncta form in response to the recruitment of ULK1. ARPE-19 cells stably expressing mitochondrially localized mCherry-FIS1[101-152] were transduced to express the indicated proteins for 24 h before immunofluorescence staining using an anti-WIPI2 and an anti-ULK1 antibody and confocal microscopy analysis. Left: Representative images. Scale bar: 5 µm. Right: Quantification of the mean number of WIPI2 puncta on mCherry-positive mitochondria from 3 independent experiments with a minimum of 25 cells being analyzed per condition in each experimental replicate. Statistics: Two-tailed unpaired t-test.


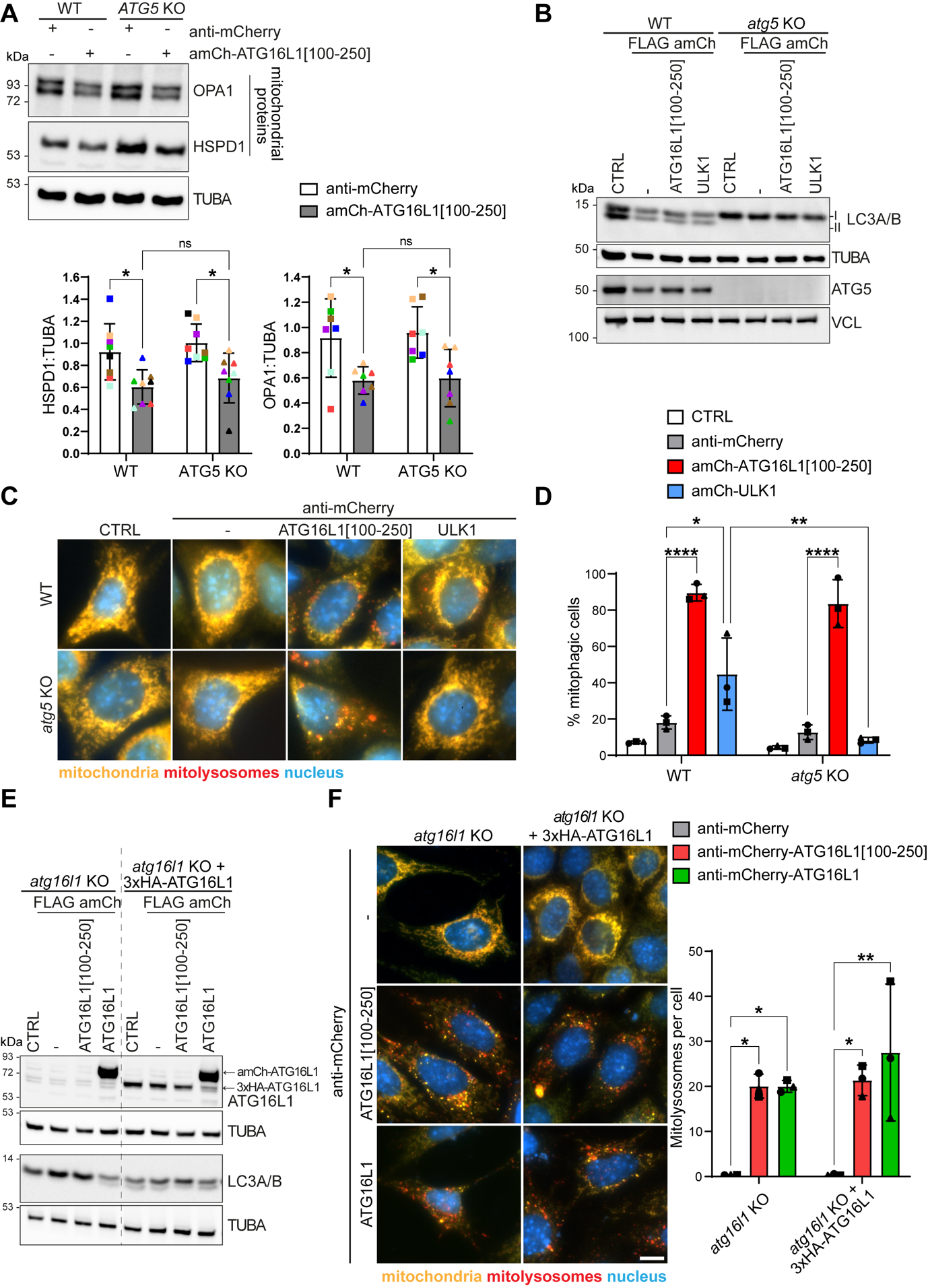


**Figure S6.** Mitophagy induction upon mitochondrial recruitment of ATG16L1[100-250] does not require ATG5 or endogenous ATG16L1. (**A**) WT or *ATG5* KO *mito*-QC A549 cells were transduced to express the indicated proteins for 48 h before cell lysis and immunoblot analysis. Displayed is a representative immunoblot and a quantitation of mitophagy showing the levels of the mitochondrial proteins HSPD1/HSP60 and OPA1 normalized to TUBA/tubulin from a minimum of 7 independent experiments. Statistics: Two-Way ANOVA + Tukey’s multiple comparisons test. (**B**) WT or *atg5* KO *mito*-QC MEFs were transduced with the indicated proteins (or not transduced [CTRL]) for 48 h before cell lysis. Shown is a representative immunoblot. (**C**) WT or *atg5* KO *mito*-QC MEFs transduced as in B, were fixed and analyzed by microscopy. Shown are representative *mito*-QC images. Scale bar: 5 µm. (**D**) Quantification of mitophagy in WT or *atg5* KO *mito*-QC MEFs, treated as in C, by flow cytometry analysis. Displayed is the quantification of mitophagy showing the mean percentage of mitophagic cells from 3 independent experiments. Statistics: Two-Way ANOVA + Tukey’s multiple comparisons test. (**E**) *atg16l1* KO or *atg16l1* KO + 3xHA-ATG16L1 (KO rescue) *mito*-QC MEFs were transduced to express the indicated ATG16L1 proteins (or were not transduced [CTRL]) for 48 h before cell lysis. Shown is a representative immunoblot. Note, the anti-ATG16L1 antibody recognizes the N-terminal part of ATG16L1 and thus does not recognize anti-mCherry-ATG16L1[100-250]. (**F**) Cells treated as in E were fixed and analyzed by microscopy. Left: Representative images. Scale bar: 10 µm. Right: Quantification of mitophagy showing the mean number of mitolysosomes per cell from 3 independent experiments with a minimum of 52 cells being analyzed per condition in each experiment. Statistics: Two-Way ANOVA + Tukey’s multiple comparisons test.


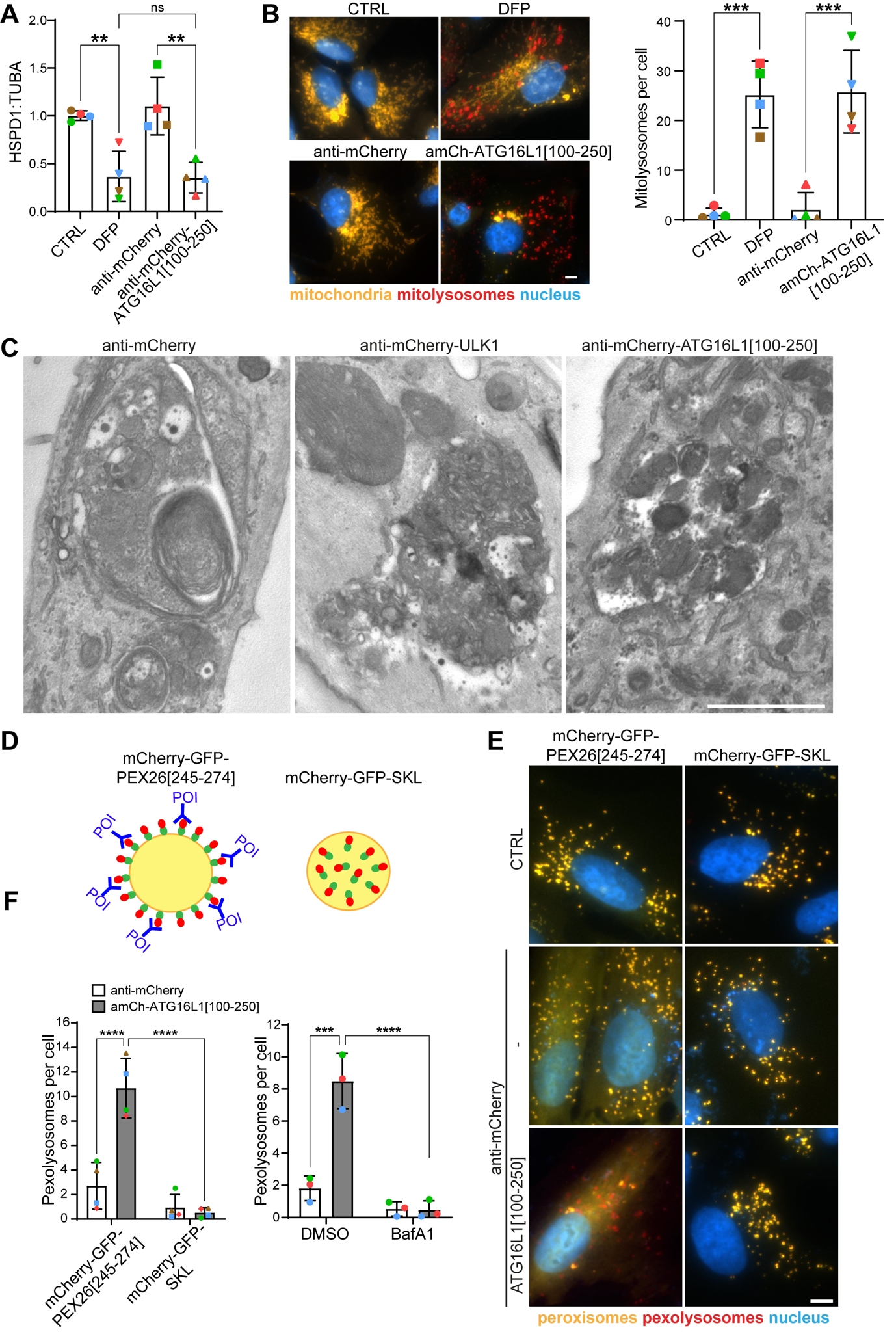


**Figure S7.** Targeting of ATG16L1[100-250] to peroxisomes induces pexophagy. (**A**) ARPE-19 *mito*-QC cells were transduced (or not [CTRL]) to express the indicated proteins, or were treated with DFP (1 mM) for 48 h before cell lysis and immunoblot analysis. Quantification of mitophagy is shown via levels of the mitochondrial protein HSPD1 normalized to Tubulin from 4 independent experiments. Statistics: One-Way ANOVA + Tukey’s multiple comparisons test. (**B**) ARPE-19 *mito*-QC cells were treated as in A before fixing and widefield microscopy analysis. Left: Representative images. Scale bar: 5 µm. Right: Quantification of mitophagy showing the mean number of mitolysosomes per cell from at least 3 independent experiments with a minimum of 33 cells being analyzed per condition in each experiment. Statistics: One-Way ANOVA + Tukey’s multiple comparisons test. Note: This experiment was performed at the same time as the experiment in Fig. 5A and hence used the same controls. (**C**) Representative electron microscopy images from cells treated as in Fig. 7D, showing enlarged early endosome-like structures. Scale bar: 1 µm. (**D**) Schematic of cell-based pexophagy reporters: mCherry-GFP-PEX26[245-274] on the peroxisomal surface (allowing the binding of the anti-mCherry nanobody) or mCherry-GFP-SKL in the peroxisomal lumen (inaccessible to the anti-mCherry nanobody). (**E**) ARPE-19 cells stably expressing either pexophagy reporter were transduced to express the indicated proteins (or were not transduced [CTRL]) for 48 h before widefield microscopy analysis. Scale bar: 5 µm. (**F**) Quantification of pexophagy showing the mean number of pexolysosomes per cell from at least 3 independent experiments with a minimum of 39 cells analyzed per condition in each experiment. Statistics: Two-Way ANOVA + Tukey’s multiple comparisons test. Separately, cells as in E were treated for the final 24 h with either BafA1 (50 nM) or DMSO (0.05%) before fixing and microscopy analysis. Quantitation of pexophagy is shown as the mean number of pexolysosomes per cell from 3 independent experiments with a minimum of 26 cells being analyzed per condition in each experiment. Statistics: Two-Way ANOVA + Tukey’s multiple comparisons test.
